# Relevance of time‐dependence for clinically viable diffusion imaging of the spinal cord

**DOI:** 10.1002/mrm.27463

**Published:** 2018-09-05

**Authors:** Francesco Grussu, Andrada Ianuş, Carmen Tur, Ferran Prados, Torben Schneider, Enrico Kaden, Sébastien Ourselin, Ivana Drobnjak, Hui Zhang, Daniel C. Alexander, Claudia A. M. Gandini Wheeler‐Kingshott

**Affiliations:** ^1^ Queen Square MS Centre, UCL Institute of Neurology, Faculty of Brain Sciences University College London London United Kingdom; ^2^ Centre for Medical Image Computing, Department of Computer Science University College London London United Kingdom; ^3^ Champalimaud Centre for the Unknown Champalimaud Foundation Lisbon Portugal; ^4^ Centre for Medical Image Computing, Department of Medical Physics and Biomedical Engineering University College London London United Kingdom; ^5^ Philips United Kingdom Guildford Surrey United Kingdom; ^6^ Clinical Imaging Research Centre National University of Singapore Singapore Singapore; ^7^ Brain MRI 3T Research Centre C. Mondino National Neurological Institute Pavia Italy; ^8^ Department of Brain and Behavioural Sciences University of Pavia Pavia Italy

**Keywords:** diffusion time, Monte Carlo simulations, spinal cord, white matter, microstructure

## Abstract

**Purpose:**

Time‐dependence is a key feature of the diffusion‐weighted (DW) signal, knowledge of which informs biophysical modelling. Here, we study time‐dependence in the human spinal cord, as its axonal structure is specific and different from the brain.

**Methods:**

We run Monte Carlo simulations using a synthetic model of spinal cord white matter (WM) (large axons), and of brain WM (smaller axons). Furthermore, we study clinically feasible multi‐shell DW scans of the cervical spinal cord (*b* = 0; *b* = 711 s mm^−2^; *b* = 2855 s mm^−2^), obtained using three diffusion times (Δ of 29, 52 and 76 ms) from three volunteers.

**Results:**

Both intra‐/extra‐axonal perpendicular diffusivities and kurtosis excess show time‐dependence in our synthetic spinal cord model. This time‐dependence is reflected mostly in the intra‐axonal perpendicular DW signal, which also exhibits strong decay, unlike our brain model. Time‐dependence of the total DW signal appears detectable in the presence of noise in our synthetic spinal cord model, but not in the brain. In WM in vivo, we observe time‐dependent macroscopic and microscopic diffusivities and diffusion kurtosis, NODDI and two‐compartment SMT metrics. Accounting for large axon calibers improves fitting of multi‐compartment models to a minor extent.

**Conclusions:**

Time‐dependence of clinically viable DW MRI metrics can be detected in vivo in spinal cord WM, thus providing new opportunities for the non‐invasive estimation of microstructural properties. The time‐dependence of the perpendicular DW signal may feature strong intra‐axonal contributions due to large spinal axon caliber. Hence, a popular model known as “stick” (zero‐radius cylinder) may be sub‐optimal to describe signals from the largest spinal axons.

## 1 INTRODUCTION

Diffusion‐weighted (DW) Magnetic Resonance Imaging (MRI)^1^, ^2^ provides unprecedented insights into tissue microstructure in vivo by exploiting the ubiquitous presence of biological water, which is used as an endogenous probe. Recent research has led to the development of promising DW MRI techniques, which disentangle distinct microstructural aspects that in turn influence the measured signals. Examples are: density,^3^, ^4^ spatial configuration,^5^, ^6^, ^7^ eccentricity^8^, ^9^ and size^10^, ^11^ of anisotropic structures; water exchange among cellular compartments^12^, ^13^; intrinsic diffusion coefficients^14^; moments of distributions of diffusion tensors (DTs),^15^ related to cell morphology; pseudo‐diffusion due to spatially incoherent blood microcirculation.^16^

Another important aspect of the DW signal in biological tissues is its dependence on the diffusion time,^17^ i.e. the time during which water molecules explore their surroundings by virtue of Brownian motion before measurements are taken. The diffusion time defines the spatial length scale^18^ to which the DW signal is mostly sensitive. The time‐dependent patterns of the DW signal carry a signature of the properties of the biological structures that restrict diffusion, and accurate knowledge of these patterns informs signal modelling.

Recently, time‐dependence has been considered in several studies focussing on brain white matter (WM), where differences between intra‐axonal and extra‐axonal behaviours have been studied.^19^, ^20^, ^21^, ^22^ However, to date much less attention has been given to the spinal cord,^23^ despite it being a small but functionally relevant structure of the central nervous system. Spinal cord WM differs from that of the brain: spinal axons can be substantially larger, with the tails of myelinated axon diameter distributions extending up to 15 μ m or beyond,^24^, ^25^, ^26^ compared to roughly 6 μ m in brain areas such as the corpus callosum.^27^ It is possible that such microstructural differences between brain and spinal cord could lead to distinct patterns of time‐dependence. Knowledge of these patterns in the spinal cord is essential to inform accurate biophysical modelling and thus obtain highly specific indices of axon morphology. These are sought in a number of conditions such as multiple sclerosis,^28^ spinal cord injury,^29^, ^30^ spondyloic myelopathy and others,^31^ given the limited prognostic value of conventional imaging readouts.

Here we characterise time‐dependent patterns of the DW signal in the human spinal cord, following previous preliminary investigation.^32^, ^33^ We ran Monte Carlo (MC) simulations of the diffusion process within geometries representative of spinal cord white matter (i.e. characterised by large axons) and analysed DW scans performed with clinically feasible protocols in vivo.

Our MC simulations allow the estimation of the intra‐ and extra‐axonal contributions to the observed time‐dependence for diffusion weighting perpendicular to the axon longitudinal axis. Moreover, they enable the evaluation of the appropriateness of a popular geometric model used to approximate intra‐axonal DW signals, referred to as the “stick”^34^, ^35^ (a zero‐radius cylinder). The “stick” model was introduced previously for practical imaging in the brain,^6^, ^7^, ^34^, ^36^ assuming negligible perpendicular intra‐axonal diffusion. Nonetheless, it is unclear to what extent such an approximation holds for the largest spinal axons, whose diameters can exceed those of the brain, and for the gradient strengths and signal‐to‐noise ratios typical of clinical hardware.^37^, ^38^, ^39^

Furthermore, this paper assesses for the first time the relevance of considering time‐dependence in clinically feasible DW imaging of the spinal cord. Inspired by recent brain studies,^22^, ^40^ we test whether time‐dependence of macroscopic^41^ and microscopic^42^ diffusion tensor imaging (DTI) parameters as well as diffusion kurtosis imaging (DKI) and two‐compartment^6^, ^36^ model parameters can be measured in the spinal cord. Also, our in vivo data enable a formal evaluation of the performance of the “stick” model in the spinal cord, by explicitly testing the impact of neglecting intra‐axonal perpendicular diffusion in terms of goodness of fit.

## 2 METHODS

### 2.1 In silico study

We ran MC simulations in Camino^43^ to characterise patterns of diffusion time‐dependence for diffusion sensitisation perpendicular to the axon longitudinal axis. Synthetic data generated with Camino were then analysed with custom‐written code in Matlab 2016b (The MathWorks, Inc., Natick, Massachusetts, USA).

#### 2.1.1 Substrates

**Figure 1:**
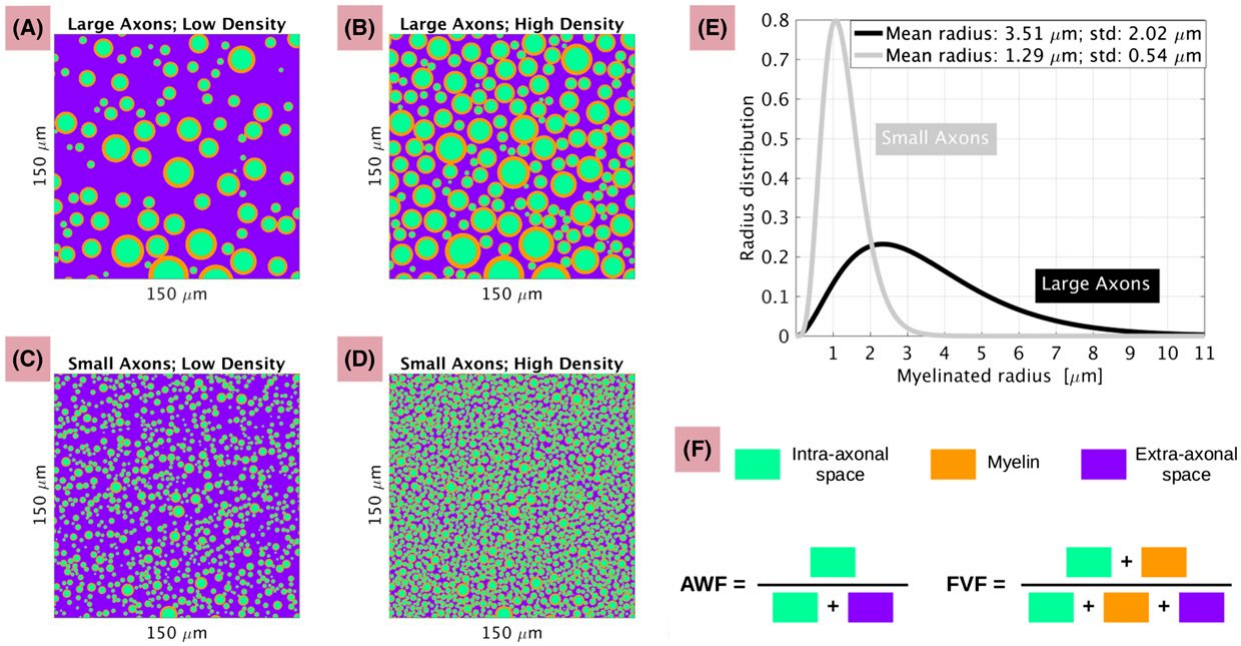
Description of the substrates used for MC simulations. Panels A‐D: illustration of a 150 μ m × 150 μ m axial detail of the 200 μ m × 200 μ m substrates (i.e. such that the normal to the sectioning plane is parallel to the cylinder longitudinal axis). Light green represents the intra‐axonal space; orange myelin; violet the extra‐axonal space. A and B illustrate the substrate with large axons (A: low axonal density; B: high axonal density); C and D the substrate with small axons (C: low axonal density; D: high axonal density). E: myelinated axon diameter distributions corresponding to the two substrates, with large and small axons. F: definition of *axonal water fraction* (AWF) and *fibre volume fraction* (FVF). AWF is the fraction of unmyelinated intra‐axonal space with respect to the total MR‐visible water (i.e. excluding myelin water due to its short T2); FVF is the volume fraction of myelinated fibres with respect to the total voxel volume. For both substrates with large and small axons, low axonal density (panels A and C) corresponds to AWF = 0.27 and FVF = 0.4; high axonal density (panels B and D) to AWF = 0.57 and FVF = 0.7

We modelled WM as a substrate made of impermeable, parallel cylinders aligned along the *z* direction, representing axons.^11^, ^21^ We studied two different types of substrates (Figure 1, panels A‐D): one characterised by larger axons, and replicating the largest spinal axons in post mortem measurements^24^, ^25^, ^26^; the other chacterised by smaller axons, and replicating a previous model of callosal WM.^11^

We described the distribution of axon radii *R* with a gamma distribution,^11^, ^20^, ^21^ parametrised by the shape (*a*; dimensionless) and the scale (*b*; in μ m) parameters. *a* and *b* relate to expected value and variance of *R* as *E*[*R*] = *a* *b* and *Var*[*R*] = *a* *b* ^2^.^35^

Here, we considered myelinated axons, and therefore used specific values for *a* and *b* to describe the distributions of outer cylinders (axons including myelin) and inner cylinders (axons without myelin), assuming a ratio of unmyelinated/myelinated axon radius (i.e. g‐ratio) of 0.75.^44^ In practice, for the outer cylinder radius distribution, we used: *a* = 3.01, *b* = 1.63 μ m for the substrate with large axons (*E*[*R*] = 3.51 μ m; *Var*[*R*] = 4.07 μ m^2^); *a* = 5.73 and *b* = 0.23 μ m for the substrate with small axons (*E*[*R*] = 1.29 μ m; *Var*[*R*] = 0.29 μ m^2^). Conversely, for the inner cylinder radius distribution, we used: *a* = 3.11, *b* = 0.86 μ m for the substrate with large axons (*E*[*R*] = 2.66 μ m; *Var*[*R*] = 2.28 μ m^2^); *a* = 5.69 and *b* = 0.17 μ m of the substrate with small axons (*E*[*R*] = 0.97 μ m; *Var*[*R*] = 0.17 μ m^2^). The distributions of myelinated axon radii are shown in panel E of Figure 1.

We considered two distinct axonal densities: one high, one low. For the high density, we adopted a *fibre volume fraction* (FVF; volume fraction of myelinated axons with respect to voxel volume^44^, ^45^) of 0.7, corresponding to an *axonal water fraction* (AWF; fraction of intra‐axonal water with respect to MR‐visible water^4^, ^46^) of 0.57. For the low density, we set FVF to 0.4, implying AWF of 0.27. To achieve the desired FVF, we used the following number of cylinders representative of myelinated axons within tissue cubes of 200 μ m per side: 565 for large axons, high density; 305 for large axons, low density; 4605 for small axons, high density; 2625 for small axons, low density.^47^

Panel F of Figure 1 illustrates the definition of FVF and AWF, which are related as:

(1) $$\text{AWF}\;=\;\frac{g^{2}\;\text{FVF}}{\;1\;+\;(g^{2}-1)\;\text{FVF}},$$

where *g*  ∈  [0; 1] is the g‐ratio.^44^

#### 2.1.2 Spin dynamics

We simulated the random walks of 44 · 10^3^ spins for each substrate and density running 11 times the spin dynamics, with each run being characterised by a unique random instantiation of the positions of the cylinders and of 4 · 10^3^ walkers at *t* = 0. We simulated 75 ms of dynamics,^47^ to probe diffusion times that can be realistically achieved in vivo with spin echo in the cord. We used 4 · 10^4^ time steps and assumed no exchange between compartments. We employed two values for the intrinsic diffusivities in both intra‐axonal (*D*_in, 0_) and extra‐axonal (*D*_ex, 0_) spaces: 1 μ m^2^ ms^−1^ and 2 μ m^2^ ms^−1^.^20^, ^21^ This was done to model two scenarios: *D*_ex, 0_ < *D*_in, 0_ and *D*_in, 0_  <  *D*_ex, 0_, since it is not completely clear yet whether intra‐axonal water is faster than extra‐axonal water, although some preliminary evidence leans towards intra‐axonal being faster.^48^, ^49^

#### 2.1.3 Synthetic diffusivity, kurtosis excess and DW signals

We studied the displacements of the random walkers perpendicular to the cylinder longitudinal axis (Δ*x*(*t*) = *x*(*t*)−*x*(0) and Δ*y*(*t*) = *y*(*t*)−*y*(0)) to evaluate time‐dependent perpendicular diffusivity and kurtosis excess in the intra‐/extra‐axonal spaces (*D*_in, ⊥_(*t*) and *K*_in, ⊥_(*t*); *D*_ex, ⊥_(*t*) and *K*_ex, ⊥_(*t*)). For our calculations, we relied on the definitions^43^, ^50^:

(2) $$D_{⊥}\left(t\right)\;\;\;=\;\;\;\frac{\;1\;}{\;4t\;}\;(\;E[\Delta x^{2}\left(t\right)+\Delta y^{2}\left(t\right)])$$

and

(3) $$K_{⊥}\left(t\right)\;\;=\;\;\frac{1}{2\pi }\;\int_{0}^{2\pi }K(\Delta x\left(t\right)\cos\left(\phi \right)\;+\;\Delta y\left(t\right)\sin\left(\phi \right))\;d\phi ,$$

where *E*[·] and *K*[·] are the mathematical expectation and kurtosis excess operators.^51^ In Equations (2) and (3), *D*_⊥_(*t*) identifies in turn *D*_in, ⊥_(*t*) (intra‐axonal diffusivity) and *D*_ex, ⊥_(*t*) (extra‐axonal diffusivity), while *K*_⊥_(*t*) identifies in turn *K*_in, ⊥_(*t*) (intra‐axonal kurtosis excess) and *K*_ex, ⊥_(*t*) (extra‐axonal kurtosis excess). The kurtosis excess can be either positive or negative, and a value exactly equal to zero indicates perfectly Gaussian diffusion.

We also calculated a long‐time tortuosity limit for *D*_ex, ⊥_(*t*) given the intrinsic diffusivity *D*_ex, 0_ as^52^:

(4) $$D_{\text{lim}}\;\;=\;\;(1-\text{FVF})\;D_{\text{ex},\;0}.$$

Values approximated with expression (4) have been historically adopted for in vivo DW MRI,^6^, ^36^ i.e. with intermediate values of axonal densities. Here we compare time‐dependent extra‐axonal perpendicular diffusivities from MC simulations to the tortuosity limit, to assess the extent of their agreement.

Given compartmental diffusivity and kurtosis, we approximated the DW signals relative to both intra (*S*_in, ⊥_(*t*,*b*)) and extra‐axonal (*S*_ex, ⊥_(*t*,*b*)) compartments for measurements perpendicular to the cylinder axis, at given *b*‐value *b* and diffusion time *t* as^50^:

(5) $$S_{\text{in},\;⊥}\left(t\;,\;b\right)\;\;\;\approx \;\;\;\text{AWF}\;\;e^{\;-bD_{\text{in},\;⊥}\left(t\right)\;+\;\frac{1}{6}\;b^{2}D_{\text{in},\;⊥}^{2}\left(t\right)\;K_{\text{in},\;⊥}\left(t\right)}$$

and

(6) $$S_{\text{ex},\;⊥}\left(t\;,\;b\right)\;\;\;\approx \;\;\;\left(1-\text{AWF}\right)\;\;e^{\;-bD_{\text{ex},\;⊥}\left(t\right)\;+\;\frac{1}{6}\;b^{2}D_{\text{ex},\;⊥}^{2}\left(t\right)\;K_{\text{ex},\;⊥}\left(t\right)}.$$

For the implementation of Equations (5) and (6), we used the mean values of *D*_in, ⊥_(*t*), *D*_ex, ⊥_(*t*), *K*_in, ⊥_(*t*), *K*_ex, ⊥_(*t*) over the random seeds. Lastly, we evaluated the total DW signal from *S*_in, ⊥_ and *S*_ex, ⊥_ as:

(7) $$S_{\text{tot},\;⊥}\left(t\;,\;b\right)\;\;\;=\;\;S_{\text{in},\;⊥}\left(t\;,\;b\right)\;+\;S_{\text{ex},\;⊥}\left(t\;,\;b\right).$$

#### 2.1.4 Effect of noise

We evaluated the effect of noise on the possibility of detecting time‐dependent signal changes in clinically realistic scenarios. For this purpose, we calculated percentage relative differences between the total signal at a diffusion time *t* > *t*_ref_ with respect to the total signal at a reference diffusion time *t*_ref_:

(8) $$\Delta S_{\text{tot},\;⊥}\left(t\;,\;b\right)\;\;\left[\%\right]\;\;\;=\;\;\;100\;\;\frac{\;\;S_{\text{tot},\;⊥}\left(t\;,\;b\right)-S_{\text{tot},\;⊥}\left(t_{\text{ref}},\;b\right)\;\;}{S_{\text{tot},\;⊥}\left(t_{\text{ref}},\;b\right)}.$$

*t*_ref_ represents the minimum diffusion time that can be plausibly probed in a clinical system for intermediate to high diffusion weighting in the spinal cord, here set to *t* = 25 ms.^53^ Equation (8) was implemented for all substrates illustrated in Figure 1, and for both cases when *D*_ex, 0_′, <  *D*_in, 0_ and *D*_in, 0_ < *D*_ex, 0_. For the evaluation of Δ*S*_tot, ⊥_(*t* , *b*)  [%] at each *t*,* S*_tot, ⊥_(*t* , *b*) and *S*_tot, ⊥_(*t*_ref_ , *b*) were corrupted with Rician noise independently for 500 times, at a realistic signal‐to‐noise ratio (SNR) of 10 at *b* = 0.^53^

### 2.2 In vivo study

We studied the relevance of considering time‐dependence for clinically feasible DW imaging in vivo. For this purpose, we acquired DW data from three healthy volunteers using a range of diffusion times.

We analysed the data to test whether diffusion time‐dependence of popular diffusion MRI metrics can be observed in the spinal cord, in view of recent findings that measured this effect in the brain.^22^, ^40^

Specifically, we considered well established DTI and DKI parameters, as well as recent microscopic DT metrics from single‐compartment spherical mean technique (SMT).^42^ DTI and DKI parameters are influenced by the orientation distribution of the axonal segments within the voxel. Conversely, microscopic DTI metrics from SMT describe the average properties of the single axonal segment, irrespective of its spatial orientation and of the overall orientation distribution of the segments.^42^

Additionally, we also considered metrics from popular multi‐compartment models, namely NODDI^6^ and two‐compartment SMT.^36^ These approaches aim to separate the intra/extra‐neurite components of the DW signal by making assumptions on the diffusion characteristics of each compartment, attempting to deal with noisy and scarce data sets as those available in clinical settings.

The next subsections describe the details of the in vivo study, namely: MRI acquisition; post‐processing of the acquired images including denoising and co‐registration; model fitting and analyses.

#### 2.2.1 MRI acquisition

We recruited three healthy subjects (1 female, 2 males; all 27 y.o.) and scanned them in sessions approved by a local research Ethics Committee, following informed written consent. We used a 3 Tesla Philips Achieva MRI system (gradient strength of 63 mT  m^−1^), with a 16‐channel neurovascular receive‐only RF coil.

On each subject, we acquired axial‐oblique MRI slices perpendicular to the cord longitudinal axis, centering the field‐of‐view (FOV) at C2‐C3. Our protocol was run within a single imaging session and consisted of a fast‐field‐echo (FFE) scan (resolution of 0.75 × 0.75 mm^2^; slice thickness of 5 mm; FOV of 240 × 180 × 60 mm^3^; TE/TR =  4.1/20  ms/ms; flip angle of 7^∘^; 4 averages) and of three clinically feasible, multi‐shell DW scans. The DW scans were performed using cardiac‐gated pulsed‐gradient spin echo (PGSE) ZOOM EPI,^53^, ^54^ with parameters: resolution 1 × 1 mm^2^; slice thickness of 5 mm; FOV of 64 × 48 × 60 mm^3^; TE of 111 ms; TR of 12 heart beats; peripheral triggering (delay: 150 ms); outer volume suppression^55^; *b*‐values of 711 s mm^−2^ (20 isotropically‐distributed directions) and 2855 s mm^−2^ (40 isotropically‐distributed directions), plus 6 interleaved *b* = 0; gradient duration *δ* of 22 ms. Each two‐shell acquisition was characterised by a unique value of gradient separation Δ among {29, 52, 76} ms, while gradient strength varied to match the *b*‐values across two‐shell protocols.

Each two‐shell diffusion scan at fixed Δ had a nominal duration of 16 min and 17 s (cardiac gated), while the FFE scan duration was of 4 min and 40 s. Overall, the whole experiment had a nominal duration of just above 55 minutes, including scout scan and calibrations.

#### 2.2.2 Image post‐processing

We post‐processed MRI scans to improve image quality and align anatomical/diffusion data, as described below:

1. we denoised the DW data using the Marčenko‐Pastur principal component analysis (MP‐PCA) algorithm from MRtrix3 (dwidenoise 56), which detects and removes noisy eigenvalues after PCA of measurement covariance matrix;
2. we mitigated Rician bias using a custom Python implementation of the method of moments^57^ and obtained an estimate of the SNR dividing the mean *b* = 0 signal by the estimated standard deviation of underlying Gaussian noise;
3. we corrected for motion the DW scans using slice‐wise linear registration with FSL flirt,^58^ as shown previously^53^, ^59^;
4. we segmented the spinal cord on the FFE scans using the Spinal Cord Toolbox (SCT)^60^ (sct_propseg 61);
5. we non‐linearly co‐registered the SCT template with the mean non‐DW image; this was done by combining with sct_concat_transfo the co‐registration transformations between FFE and mean non‐DW images (obtained with sct_register_multimodal) and between FFE and SCT template (obtained with sct_register_to_template)^60^;
6. for visualisation and quality assurance, we also warped the atlases defined in the SCT template space to the DW image space with SCT sct_warp_template.

For each subject and fixed value of Δ, we fitted voxel‐by‐voxel a number of signal models that hold promise for clinical translation and application in neurological disorders, namely DTI, microscopic DTI via SMT, DKI, NODDI and two‐compartment SMT. Table 1 summarises the metrics that were considered in this study.

**Table 1:** Summary of the metrics obtained in vivo and compared across diffusion times with mixed effects models

Metric	Model	Meaning
AD_DTI_	Macroscopic DTI	Parallel diffusivity of macroscopic diffusion tensor
RD_DTI_	Macroscopic DTI	Perpendicular diffusivity of macroscopic diffusion tensor
*μ*AD	Microscopic DTI	Parallel diffusivity of per‐axon diffusion tensor
*μ*RD	Microscopic DTI	Perpendicular diffusivity of per‐axon diffusion tensor
AD_DKI_	Macroscopic DKI	Parallel diffusivity of macroscopic diffusion tensor
RD_DKI_	Macroscopic DKI	Perpendicular diffusivity of macroscopic diffusion tensor
AK_DKI_	Macroscopic DKI	Parallel kurtosis of macroscopic kurtosis tensor
RK_DKI_	Macroscopic DKI	Perpendicular kurtosis of macroscopic kurtosis tensor
V_NODDI_	NODDI	Voxel volume fraction of neurite compartment
ODI	NODDI	Dispersion of neurite orientation distribution
V_SMT_	Two‐compartment SMT	Voxel volume fraction of neurite compartment
H	Two‐compartment SMT	Relative entropy of neurite orientation distribution
D	Two‐compartment SMT	Parallel diffusivity in intra‐/extra‐neurite compartments

#### 2.2.3 Voxel‐wise fitting

We performed well‐established DTI^41^ and DKI^62^ but also considered novel, clinically‐feasible approaches such as single‐compartment SMT.^42^ In WM, DTI and DKI provide metrics that depend on the spatial configuration of the axonal segments within the voxel, thus conflating orientation and microstructural effects. On the other end, single‐compartment SMT provides a first‐order descriptor (i.e. a DT) of the average properties of the individual axonal segment within a voxel, factoring out the confounding effects of axonal orientation distributions. Therefore, single‐compartment SMT provides a “microscopic” DT, which is independent of the spatial arrangements of the segments and is a marker of microscopic tissue structure. To better highlight this, we will refer to the diffusion and kurtosis tensors from DTI and DKI as “macroscopic”, while we will refer to the per‐axon diffusion tensor from SMT as “microscopic.”

We estimated the macroscopic DT with conventional DTI analysis^41^ (FSL dtifit). The DTI model was fitted to the measurements obtained at *b* = 0 and *b* = 711 s mm^−2^, e.g. excluding measurements at high *b*, where departures from Gaussianity are higher. Conversely, the microscopic DT was estimated using SMT^42^ (http://github.com/ekaden/smt). Macroscopic DKI metrics were instead obtained from constrained weighted linear least squares fitting (https://github.com/NYUDiffusionMRI/DiffusionKurtosisImaging)^62^.

From the calculated tensors, we evaluated axial and radial diffusivity (macroscopic AD_DTI_ and RD_DTI_ from DTI; macroscopic AD_DKI_ and RD_DKI_ from DKI; microscopic *μ*AD and *μ*RD from SMT) and axial and radial kurtosis (macroscopic AK_DKI_ and RK_DKI_ from DKI). The metrics are summarised in Table 1).

Additionally, we also fitted voxel‐by‐voxel two models that describe the total DW signal as arising from two non‐exchanging Gaussian compartments,^4^, ^6^, ^7^, ^34^, ^36^, ^48^ thought to be representative of intra‐/extra‐axonal spaces. Specifically, we fitted NODDI^6^ and two‐compartment SMT,^36^ e.g. two popular methods that provide summary information on water compartmentalisation in WM from noisy and scarce data sets as those typically available in clinical settings. For model fitting, freely available code was used (NODDI: http://mig.cs.ucl.ac.uk/index.php?n=Download.NODDI; SMT: http://github.com/ekaden/smt).

#### 2.2.4 Diffusion time‐dependence assessment

We tested whether quantitative metrics obtained from conventional DTI, microscopic DTI with SMT, DKI, NODDI and two‐compartment SMT (see Table 1) show significant dependence on the choice of the diffusion time, i.e. as the gradient separation Δ varied from 29 to 76 ms.

Firstly, we warped all metrics from all subjects and values of Δ to the SCT template and atlas space, using the previously estimated non‐linear registration transformations. Secondly, we extracted voxel‐wise values of the warped metrics within two distinct WM regions‐of‐interest (ROIs), corresponding to motor and sensory WM. The two ROIs excluded the top and bottom MRI slices due to post‐processing‐induced image degradation, and were obtained by thresholding the SCT WM atlas (maximum partial volume of 20%). The two ROIs included the following WM tracts: lateral corticospinal tract, rubrospinal tract, vestibulospinal tract, ventral corticospinal tract, tectospinal tract for motor WM; fasciculus gracilis, fasciculus cuneatus, ventral and dorsal spinocerebellar tracts, spino‐olivary tract, spinal lemniscus for sensory WM. Finally, we assessed diffusion time‐dependence by fitting random intercept and random coefficient mixed effects models where the relevant diffusion metric (measured at each voxel) was considered as the dependent variable and Δ as the explanatory variable. These models had three levels of hierarchy: the variable Δ was nested in a voxel identifier; then, the voxel identifier was nested in a subject identifier. Overall, our approach robustly pools information from multiple voxels, diffusion times and subjects, while accounting for correlations within data points (e.g. within the same voxel across diffusion times as well as within multiple voxels from the same subject). The models provide estimates of $\frac{dm}{d\Delta }$, where *m* indicates a generic diffusion metric. The estimated variation of each metric *m* for an increase of Δ of 47 ms (from 29 to 76 ms, i.e. quantity $\frac{dm}{d\Delta }\;\times \;47\;\text{ms}$) was converted to a percentage change with respect to baseline values at Δ = 29 ms. Such baseline values were chosen as the ROI‐wise medians of the average population maps in SCT template space.

#### 2.2.5 Model comparison

We tested whether accounting for a finite perpendicular diffusivity for the intra‐axonal signal (e.g. rather than a “stick”) can be beneficial for model fitting. For this purpose, we fitted two multi‐compartment models of the DW signal to the multi‐shell set of measurements at fixed Δ and compared the goodness of fit. Similarly to NODDI and two‐compartment SMT, these models describe the total signal as a linear combination of those from two non‐exchanging Gaussian compartments,^4^, ^6^, ^7^, ^34^, ^36^, ^48^ representative of intra‐/extra‐axonal spaces. However, for this experiment we do not adopt the tortuosity model for extra‐neurite diffusion, and fit for compartment‐specific intrinsic diffusion coefficients.

In the first model, referred to as “Zeppelin‐stick” (ZepStick), the intra‐axonal compartment has a volume fraction *v*_*s*_ and is described by a rotationally symmetric tensor with principal diffusivity *D*_*s*, ‖_ and zero perpendicular diffusivity (a “stick”), while the extra‐axonal compartment is described by an axially symmetric tensor (i.e. a “Zeppelin”^35^), whose principal eigenvalue equals *D*_*z*, ‖_ and whose second/third eigenvalues equal *D*_*z*, ⊥_ (*D*_*z*, ⊥_≤*D*_*z*, ‖_).

The second model, referred to as “Zeppelin‐Zeppelin” (ZepZep), is a generalisation of ZepStick in that the intra‐axonal tensor has a small but non‐vanishing perpendicular diffusivity *D*_*s*, ⊥_, satisfying *D*_*s*, ⊥_ ≤ *D*_*z*, ⊥_.

Formally, the DW signal is modelled as

(9) $$S\;\;\;=\;\;\;S_{0}\;(v_{s}\;e^{-b(D_{s,⊥}\;+\;\left(D_{s,\;\|}-D_{s,⊥}\right)\;{\left(g\;\cdot \;n\right)}^{2})}\;\;\;+\;\;\;\left(1-v_{s}\right)\;e^{-b(D_{z,⊥}\;+\;\left(D_{z,\;\|}-D_{z,⊥}\right)\;{\left(g\;\cdot \;n\right)}^{2})})$$

In Equation (9), *S*_0_, **g** and **n** are respectively the DW‐signal and the gradient and main fibre directions; the subscripts *s*/*z* indicate parameters from the “stick”/“Zeppelin”. To recapitulate, *D*_*s*, ⊥_ = 0 for model ZepStick and *D*_*s*,⊥_≤*D*_*z*,⊥_ for model ZepZep.

In our implementation, we required that *D*_*z*, ‖_≤*D*_*s*, ‖_, since recent evidence suggests that intra‐axonal water (i.e. the “stick‐like” water pool) may diffuse faster inside rather than outside axons.^48^, ^49^, ^63^ Also, we fixed **n** to the principal direction of the previously estimated DT, and estimated the other model parameters via likelihood maximisation, implemented in Python as a two‐stage procedure (objective function minimisation initialised by a grid search^6^, ^35^).

We compared the quality of fit of the ZepStick and ZepZep models running a voxel‐wise likelihood ratio test with Matlab 2016b in each subject and at any fixed value of Δ. The test assesses whether an increase in likelihood provided by a more complex model is paid off by increased model complexity (i.e. it accounts for differences in the number of parameters between the two models). The test is designed for those cases when one model is a special case of another, as it happens here with ZepStick, a special case of ZepZep when *D*_*s*, ⊥_ = 0.

## 3 RESULTS

### 3.1 In silico study

#### 3.1.1 Synthetic diffusivity, kurtosis excess and DW signals

**Figure 2:**
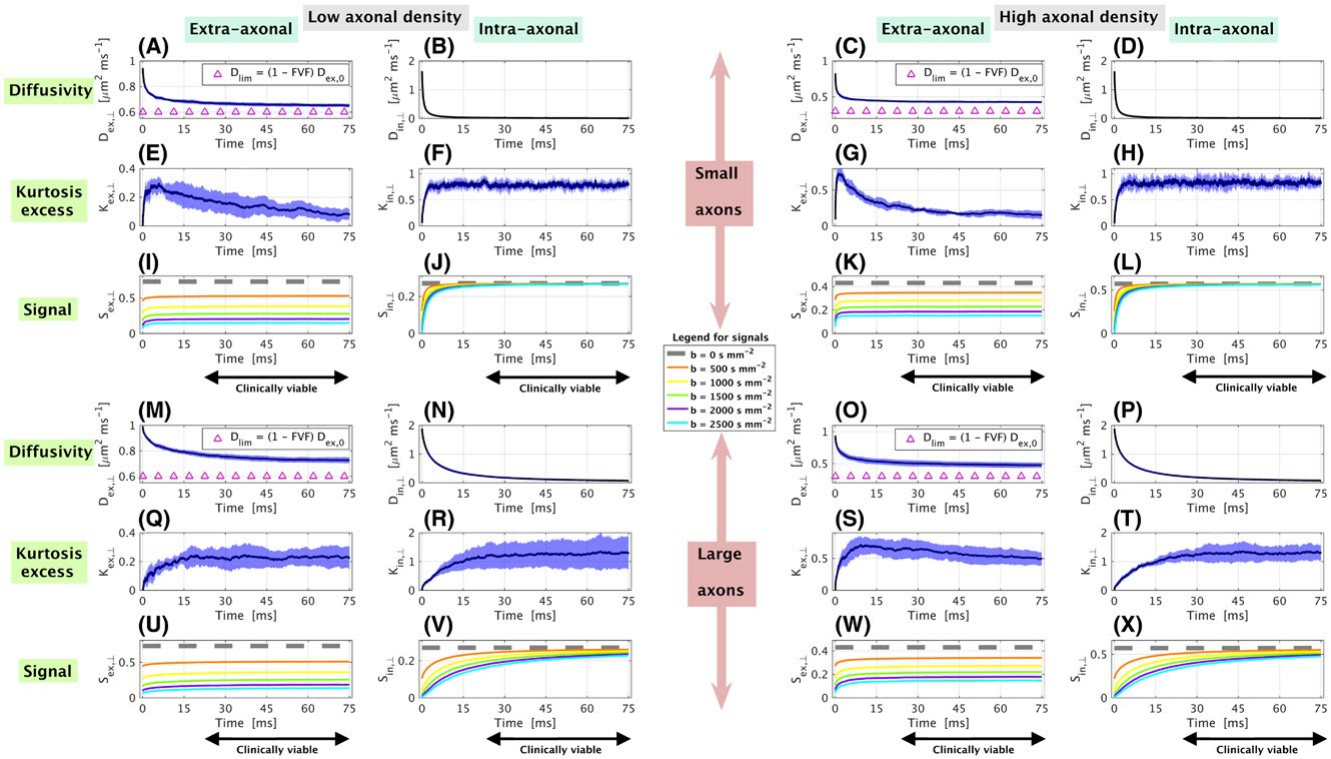
Time‐dependent perpendicular diffusivities, kurtosis excess and DW signals characterising the intra‐axonal and extra‐axonal compartments when *D*_ex, 0_ = 1 μ m^2^ ms^−1^ and *D*_in, 0_ = 2 μ m^2^,ms^−1^ (i.e. intra‐axonal water is faster than extra‐axonal water). The first two columns from the left refer to substrates with low axonal densities (extra‐axonal properties in panels A, E, I, M, Q, U; intra‐axonal properties in panels B, F, J, N, R, V), while the last two columns from the left refer to substrates with high axonal density (extra‐axonal properties in panels C, G, K, O, S, W; intra‐axonal properties in panels D, H, L, P, T, X). The first three rows from top show information for substrates with small axons (A to L), while the last three rows from top show information for substrates with large axons (M to X). The first and fourth rows from top show time‐dependent diffusivities (A to D and M to P), with the tortuosity limit $D_{\text{lim}}\;\;=\;\;(1-\text{FVF})\;D_{\text{ex},\;0}$ reported explicitly for extra‐axonal diffusivities (magenta triangles in A, C, M, O); the second and fifth rows from top show time‐dependent kurtosis excess (E to H and Q to T); the third and sixth rows provide DW signals (I to L and U to X). The plots of diffusivities and kurtosis excess report mean and standard deviation over the 11 random seeds respectively in black and light blue shade. The plots of the DW signals report signals obtained at different *b*‐values, obtained using the average diffusivity and kurtosis excess over the random seeds. Black arrows indicate diffusion times that can be probed with clinically viable acquisitions

Synthetic perpendicular diffusivities (*D*_in, ⊥_(*t*), *D*_ex, ⊥_(*t*)), kurtosis excess (*K*_in, ⊥_(*t*), *K*_ex, ⊥_(*t*)) and DW signals (*S*_in, ⊥_(*t*,*b*) and *S*_ex, ⊥_(*t*,*b*)) are shown as a function of the diffusion time *t* in Figure 2 for *D*_ex, 0_ = 1 μ m^2^ ms^−1^ and *D*_in, 0_ = 2 μ m^2^ ms^−1^, and in Supporting Information Figure S1 for *D*_ex, 0_ = 2 μ m^2^ ms^−1^ and *D*_in, 0_ = 1 μ m^2^ ms^−1^.

Firstly, we observe a marked diffusion time‐dependence of intra‐axonal and extra‐axonal perpendicular diffusivities for all substrates and densities. Importantly, most of the variation takes place in diffusion times that are unlikely to be probed using clinical systems (i.e. smaller than 25 ms). For instance, *D*_ex, ⊥_ and *D*_in, ⊥_ decrease with increasing time, and reach an asymptotic value at times that varies from case to case. When *D*_ex, 0_ < *D*_in, 0_ (Figure 2), *D*_ex, ⊥_ plateaus at about *t* = 30 ms for the small axons and at about *t* = 60 ms for the large axons. Conversely, *D*_in, ⊥_ plateaus for *t* as little as *t* = 5 ms for the small axons, while it just reaches its asymptotic values for the longest times considered here when looking at the substrate with large axons. When *D*_in, 0_ < *D*_ex, 0_ (Supporting Information Figure S1), similar behaviours of *D*_in, ⊥_ and *D*_ex, ⊥_ are observed, although in this case at the longest values of *t*,* D*_in, ⊥_ of the substrate with large axons is even further from reaching its asymptotic value. In all cases, *D*_ex, ⊥_ long‐time limits are in generally higher than the tortuosity limit $(1-\text{FVF})\;D_{\text{ex},\;0}$.

Secondly, clear time‐dependence of compartmental perpendicular kurtosis excess is also apparent. *K*_ex, ⊥_ increases sharply as the diffusion time increases from 0 to about 5 ms, and afterwards it either reaches a limit (large axons, low axonal density) or continues decreasing (all other cases, where the long‐time limit has not been reached yet). In contrast, *K*_in, ⊥_ increases as a function of time and never decreases. It reaches its long‐time limit very rapidly for small axons (in less than 10 ms for all values of *D*_in, 0_) and less rapidly for large axons (in as long as 60 ms). Moreover, the long‐time limits for the intra‐axonal perpendicular kurtosis *K*_in, ⊥_ differ from the values that one would expect for a single cylinder (*K*_in, ⊥_ = −0.5). Finally, we report that diffusion is in general more Gaussian outside rather than inside axons (*K*_ex, ⊥_ always closer to 0 than *K*_in, ⊥_), and, for the extra‐axonal compartment, it is more Gaussian in presence of small axons rather than in presence of large axons for all diffusion times.

The time‐dependence of the intra‐/extra‐axonal diffusivities and kurtosis is reflected by the corresponding DW signals *S*_in, ⊥_ and *S*_ex, ⊥_. Generally, for any strength of the diffusion weighting *b* and in both scenarios *D*_ex, 0_ < *D*_in, 0_ and *D*_in, 0_ < *D*_ex, 0_, *S*_in, ⊥_ and *S*_ex, ⊥_ show sharp increases as *t* increases from 0 to about 5 ms at fixed *b*. For higher values of *t*, changes in signal for increasing *t* are less sharp. *S*_ex, ⊥_ is practically constant for *t* bigger than about 30 ms when *D*_ex, 0_ < *D*_in, 0_ and for *t* bigger than about 20 ms when *D*_in, 0_ < *D*_ex, 0_, in all substrates (small and large axons) and for all densities. Conversely, *S*_in, ⊥_ of the small axons is practically constant for *t* bigger than about 30 ms in both cases *D*_ex, 0_ < *D*_in, 0_ and *D*_in, 0_ < *D*_ex, 0_, while *S*_in, ⊥_ of the large axons always shows marked time‐dependence for the entire range of times considered here.

Finally, the simulations show that the intra‐axonal perpendicular signal *S*_in, ⊥_ plateaus to a value that is close to the non‐DW signal for the small axons, while it shows considerable attenuation for any diffusion time *t* for the substrate with large axons and all intrinsic diffusivity scenarios. Notably, this attenuation can be as strong as 40 % of the non‐DW signal level for *b* = 2500 s mm^−2^ and *t* of the order of 30 ms.

#### 3.1.2 Effect of noise

**Figure 3:**
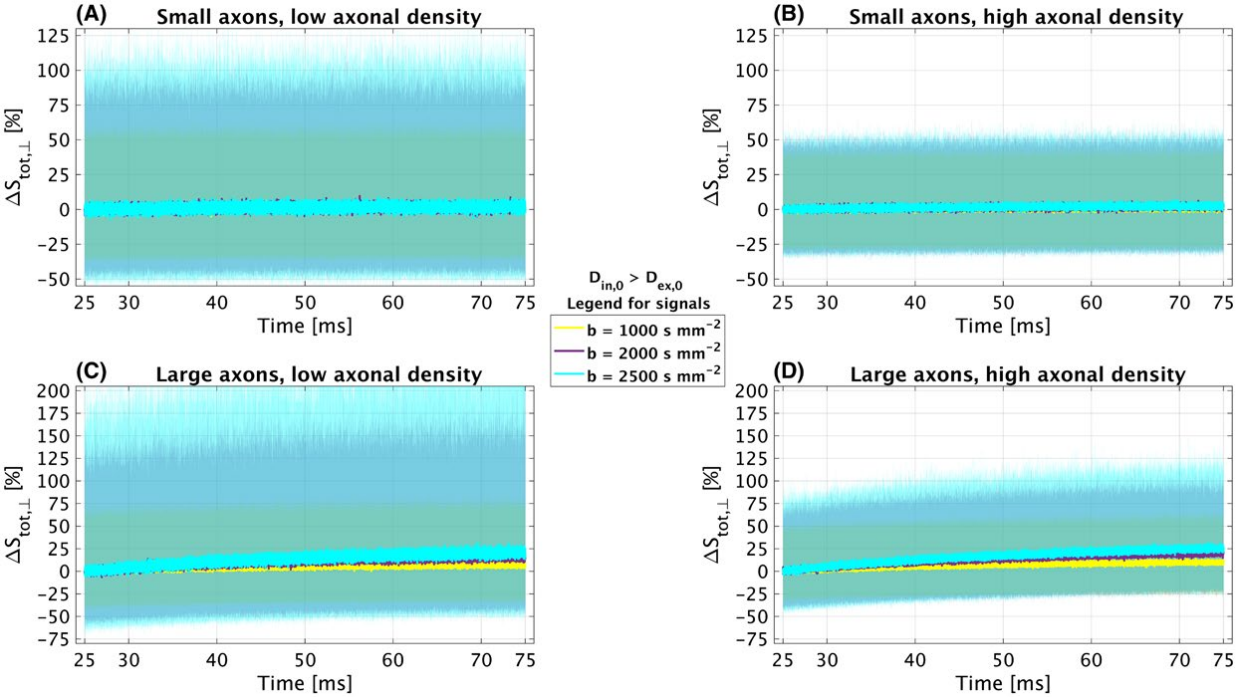
Time‐dependent patterns of Δ*S*_tot, ⊥_(*t* , *b*)  [%], defined as the percentage relative differences between the total DW signal at a diffusion time *t* with respect to the total signal at a reference diffusion time *t* = *t*_ref_ = 25 ms. The figure reports in yellow, violet and cyan values of Δ*S*_tot, ⊥_(*t* , *b*)  [%] obtained respectively at *b* = {1000, 2000, 2500} s mm^−2^, for the four synthetic substrates (small/large axons; low/high axonal density) when *D*_in, 0_ = 2 μ m^2^ ms^−1^ and *D*_ex, 0_ = 1 μ m^2^,ms^−1^ (i.e. intra‐axonal water is faster than extra‐axonal water). Solid lines report median values over 500 independent noise instantiations, with 95% of the distributions over the instantiations reported as a transparent background underneath

Figure 3 shows percentage relative signal changes at various *b*‐values with respect to a reference diffusion of 25 ms, e.g. representative of the shortest diffusion times that can be probed with standard diffusion encoding in the spinal cord within clinical settings. The figure refers to the case when *D*_ex, 0_ < *D*_in, 0_.

Figure 3 shows that for small axons increases of diffusion time from 25 ms to up to 75 ms on average do not cause appreciable changes of the total DW signal (median change indistinguishable from zero). Contrarily, for large axons and in both cases of high/low axonal densities, similar increases in diffusion time lead to detectable increases of the total DW signal in presence of noise, with a median change of up to roughly 25%. However, it should be also noted that in a fraction of voxels noise induces apparent decreases of the DW signal as the diffusion time increases, as confidence intervals in light colour cross zero.

Similar trends are observed for *D*_in, 0_ < *D*_ex, 0_ as reported in Supporting Information Figure S2.

### 3.2 In vivo study

#### 3.2.1 MRI acquisition

**Figure 4:**
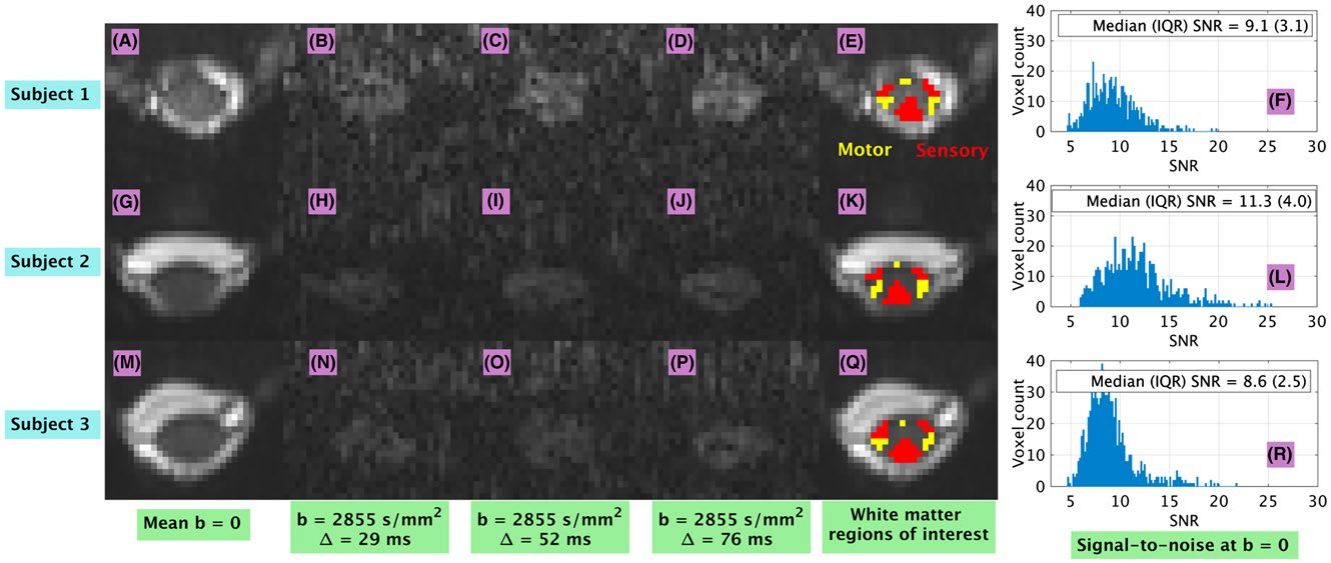
Examples of DW images and white matter ROIs. Different rows refer to different subjects (first row, panels A to F: subject 1; second row, panels G to L: subject 2; third row, panels M to R: subject 3). First column from left: mean *b* = 0 image (panels A, G, M); second to fourth column from left: example of DW images obtained for a gradient direction roughly perpendicular to the cord longitudinal axis at various diffusion times (Δ of 29 ms in panels B, H, N; Δ of 52 ms in panels C, I, O; Δ of 76 ms in panels D, J, P); fifth column from left: location of motor (in yellow) and sensory (in red) WM ROIs overlaid onto the mean *b* = 0 image (panels E, K, Q); sixth and last column from right: whole‐cord distributions of SNR at *b* = 0 (panels F, L, R, also reporting median and interquartile range of the distributions)

Figure 4 shows examples of DW images, WM ROIs and trends in SNR. The figure reveals that at *b* = 0, the median SNR within the spinal cord is on the order of 10. It also highlights a trend towards signal increase on the DW images as Δ increases, and elucidates the location of motor and sensory WM, respectively in red and yellow in panels E, K and Q.

#### 3.2.2 Voxel‐wise fitting

**Figure 5:**
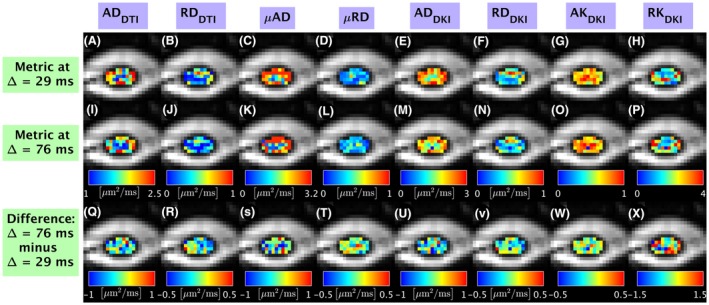
Examples of voxel‐wise metrics from single‐compartment models from subject 2 obtained at two different diffusion times, and illustrations of their voxel‐wise difference. Columns report information from different metrics. From left to right: AD_DTI_ (panels A, I, Q); RDDTI (panels B, J, R); *μ*AD (panels C, K, S); *μ*RD (panels D, L, T); AD_DKI_ (panels E, M, U); RD_DKI_ (panels F, N, V); AK_DKI_ (panels G, O, W); RK_DKI_ (panels H, P, X). The first and second rows from top respectively show metrics obtained at Δ = 29 ms (panels A to H) and Δ = 76 ms (panels I to P); the bottom row shows the difference between the two (panels Q to X)

**Figure 6:**
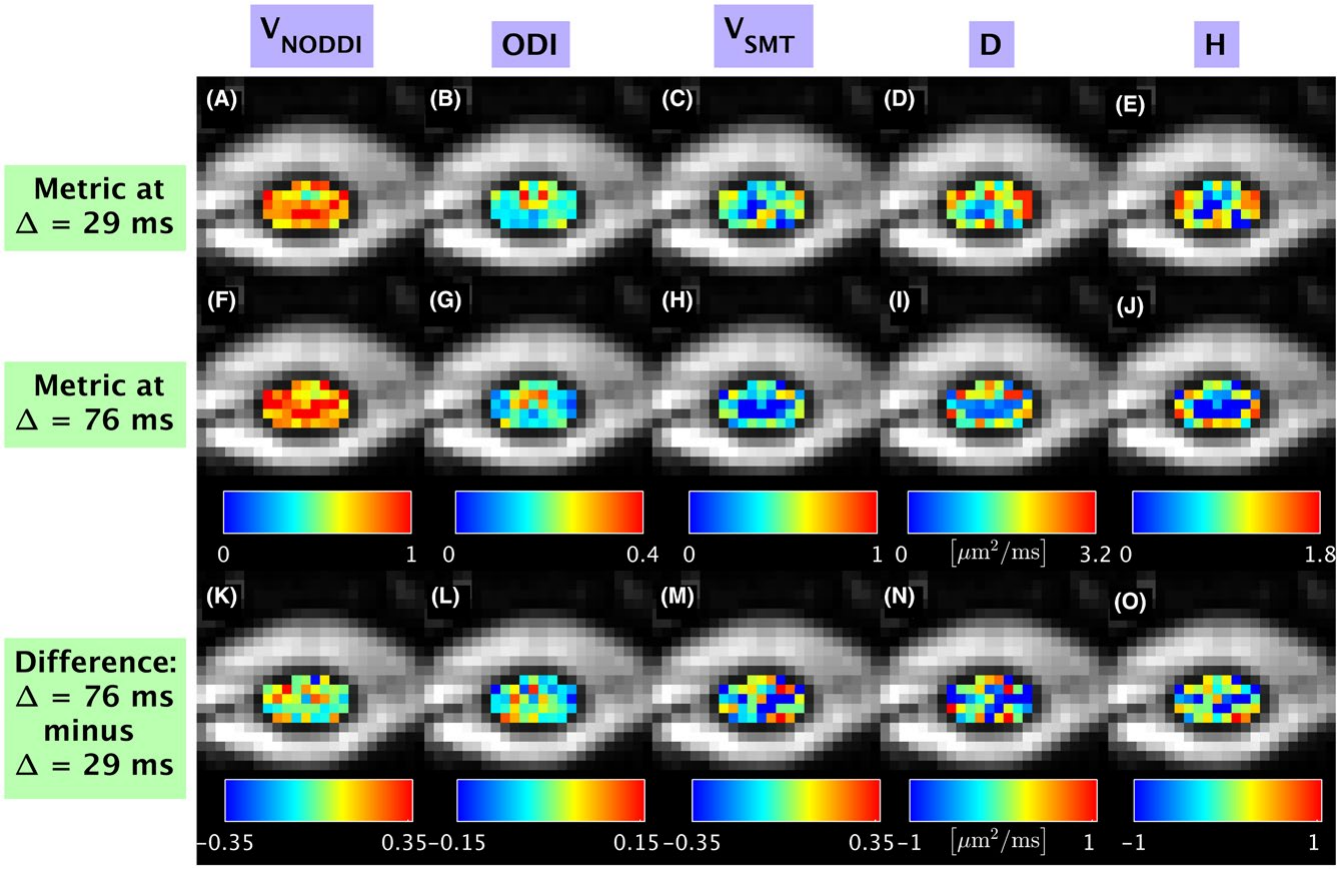
Examples of voxel‐wise metrics from two‐compartment models from subject 2 obtained at two different diffusion times, and illustrations of their voxel‐wise difference. Columns report information from different metrics. From left to right: NODDI V_NODDI_ (panels A, F, K); NODDI ODI (panels B, G, L); SMT V_SMT_ (panels C, H, M); SMT D (panels D, I, N); SMT H (panels E, J, O). The first and second rows from top respectively show metrics obtained at Δ = 29 ms (panels A to E) and Δ = 76 ms (panels F to J); the bottom row shows the difference between the two (panels K to O)

Figures 5 and 6 shows examples of voxel‐wise maps from subject 2 obtained at two diffusion times (Δ = 29 ms and Δ = 76 ms), as well as their difference (Δ = 76 ms minus Δ = 29 ms). On visual inspection, diffusivities (especially axial diffusivities AD_DTI_, AD_DKI_ and *μ*AD) are lower at Δ =  76 ms than at Δ =  29 ms. On the other hand, axial kurtosis (AK_DKI_) and radial kurtosis (RK_DKI_) increase with increasing diffusion time. Figure 6 shows quantitative maps from two‐compartment NODDI and SMT fitting from the same subject and location as Figure 5. As Δ increases from 29 to 76 ms, we observe a trend towards increase in NODDI intra‐neurite voxel volume fraction V_NODDI_ and decrease of NODDI ODI. V_SMT_ and entropy H from two‐compartment SMT show less obvious dependency on diffusion time on visual inspection, while the intrinsic neural diffusivity D decreases as Δ increases. In general, values of intra‐neurite volume fraction from SMT (V_SMT_) appear lower than values from NODDI (V_NODDI_).

#### 3.2.3 Diffusion time‐dependence assessment

Tables 2 and 3 show the estimated dependency of the diffusion metrics to changes in the gradient separation Δ (i.e. to changes in diffusion time), reported as coefficients of the mixed effects models. In both motor and sensory WM, all diffusivities from macro/microscopic DTI, DKI and two compartment SMT decrease as Δ increases, as well as NODDI ODI (negative coefficients). Axial and radial kurtosis AK_DKI_ and RK_DKI_ increase, and so does NODDI V_NODDI_ (positive coefficients). Two‐compartment SMT metrics V_SMT_ and entropy H also exhibit changes as a function of the diffusion time. Specifically, both V_SMT_ and H increase in motor WM (positive coefficients), while they both decrease (negative coefficients) in sensory WM. The strongest changes are observed for radial diffusivities (decreases of up to roughly 25%), while the weakest changes for NODDI and two‐compartment SMT volume fractions.

**Table 2:** Results from the statistical analysis evaluating diffusion time‐dependence of quantitative metrics in motor white matter. CI stands for *confidence interval*. All *p*‐values associated to the coefficients $\frac{dm}{d\Delta }$ are such that *p* < 0.001. The fifth column from left to right shows the estimated percentage variation of each metric for a change of Δ from 29 to 76 ms

	$\frac{dm}{d\Delta }$	$\frac{dm}{d\Delta }$	$\frac{dm}{d\Delta }$	*m* change for Δ
**Metric *m***	estimate	95% CI	units	from 29 to 76 ms
AD_DTI_	−2.61 · 10^−3^	[−2.80; −2.42] · 10^−3^	μ m^2^ ms^−2^	−4.9 %
RD_DTI_	−2.88 · 10^−3^	[−3.01; −2.75] · 10^−3^	μ m^2^ ms^−2^	−25.0 %
*μ*AD	−1.14 · 10^−3^	[−1.29; −0.98] · 10^−3^	μ m^2^ ms^−2^	−1.9 %
*μ*RD	−1.72 · 10^−3^	[−1.90; −1.54] · 10^−3^	μ m^2^ ms^−2^	−18.9 %
AD_DKI_	−3.48 · 10^−3^	[−4.05; −2.92] · 10^−3^	μ m^2^ ms^−2^	−5.7 %
RD_DKI_	−2.03 · 10^−3^	[−2.23; −1.82] · 10^−3^	μ m^2^ ms^−2^	−14.9 %
AK_DKI_	4.13 · 10^−4^	[3.62; 4.63] · 10^−4^	ms^−1^	3.4 %
RK_DKI_	5.03 · 10^−3^	[4.60; 5.46] · 10^−3^	ms^−1^	15.0 %
V_NODDI_	5.77 · 10^−4^	[5.30; 6.23] · 10^−4^	ms^−1^	4.1 %
ODI	−5.69·10^−4^	[−6.09;−5.29] · 10^−4^	ms^−1^	−16.0 %
V_SMT_	1.69 · 10^−4^	[0.97; 2.42] · 10^−4^	ms^−1^	2.0 %
H	7.70 · 10^−4^	[6.21; 9.19] · 10^−4^	ms^−1^	3.7 %
D	−2.86 · 10^−3^	[−3.10; −2.62] · 10^−3^	μ m^2^ ms^−2^	−5.4 %

**Table 3:** Results from the statistical analysis evaluating diffusion time‐dependence of quantitative metrics in sensory white matter. CI stands for *confidence interval*. All *p*‐values associated to the coefficients $\frac{dm}{d\Delta }$ are such that *p* < 0.001. The fifth column from left to right shows the estimated percentage variation of each metric for a change of Δ from 29 to 76 ms

	$\frac{dm}{d\Delta }$	$\frac{dm}{d\Delta }$	$\frac{dm}{d\Delta }$	*m* change for Δ
**Metric *m***	estimate	95% CI	units	from 29 to 76 ms
AD_DTI_	−4.20 · 10^−3^	[−4.33; −4.06] · 10^−3^	μ m^2^ ms^−2^	−7.8 %
RD_DTI_	−2.57 · 10^−3^	[−2.66; −2.48] · 10^−3^	μ m^2^ ms^−2^	−26.0 %
*μ*AD	−1.92 · 10^−3^	[−2.02; −1.81] · 10^−3^	μ m^2^ ms^−2^	−3.2 %
*μ*RD	−2.16·10^−3^	[−2.28; −2.04] · 10^−3^	μ m^2^ ms^−2^	−28.3 %
AD_DKI_	−6.40 · 10^−3^	[−6.85; −5.95] · 10^−3^	μ m^2^ ms^−2^	−10.6 %
RD_DKI_	−2.17·10^−3^	[−2.33;−2.01]·10^−3^	μ m^2^ ms^−2^	−18.5 %
AK_DKI_	6.15 · 10^−4^	[5.82; 6.49] · 10^−4^	ms^−1^	5.1 %
RK_DKI_	4.09 · 10^−3^	[3.82; 4.35] · 10^−3^	ms^−1^	12.4 %
V_NODDI_	5.09 · 10^−4^	[4.79; 5.39] · 10^−4^	ms^−1^	3.7 %
ODI	−5.38 · 10^−4^	[−5.65; −5.10] · 10^−4^	ms^−1^	−15.0 %
V_SMT_	−4.57 · 10^−4^	[−5.07; −4.07] · 10^−4^	ms^−1^	−5.1 %
H	−1.01 · 10^−3^	[−1.11; −0.90] · 10^−3^	ms^−1^	−5.0 %
D	−5.99·10^−3^	[−6.16; −5.81] · 10^−3^	μ m^2^ ms^−2^	−11.5 %

#### 3.2.4 Model comparison

**Figure 7:**
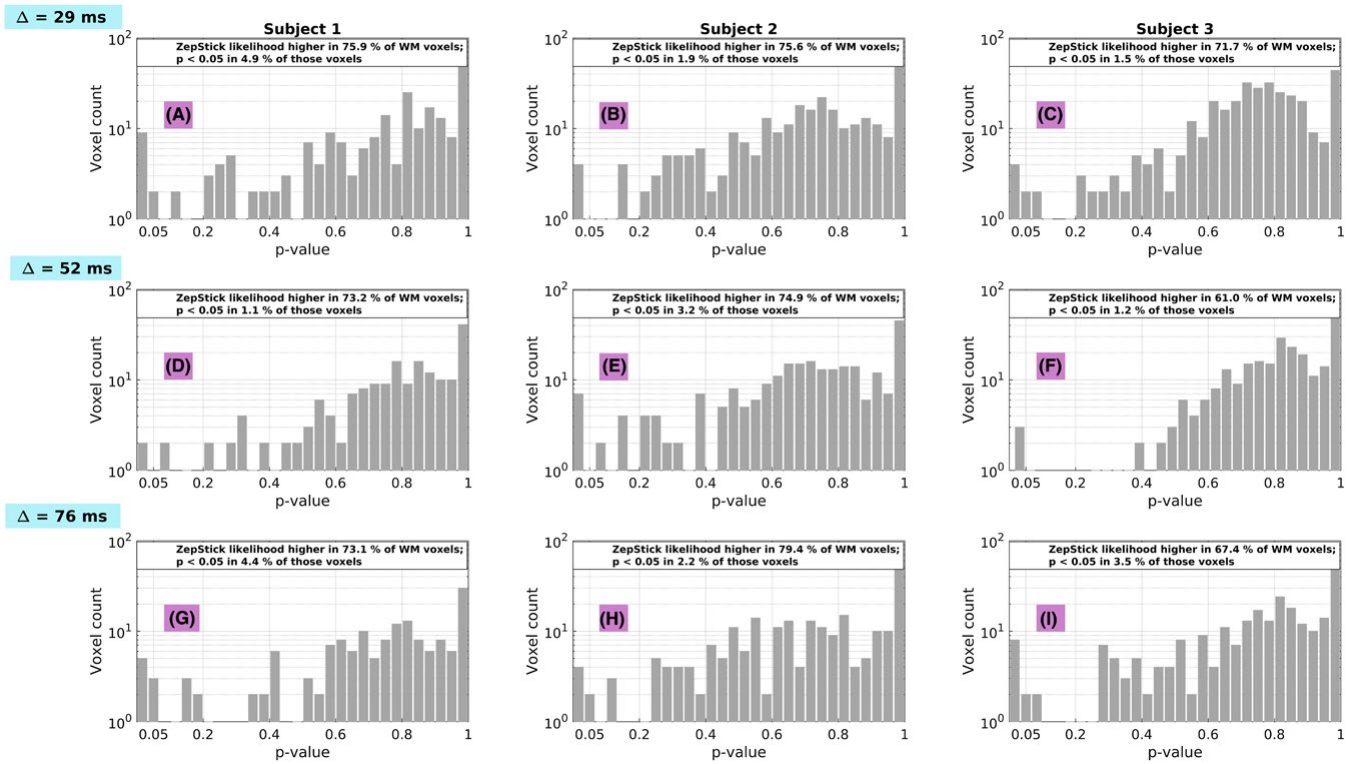
Results from the likelihood ratio test comparing the quality of fit of models ZepStick (Zeppelin‐stick) and ZepZep (Zeppelin‐Zeppelin) in all subjects and for all values of Δ (29, 52 and 76 ms). In each panel, the fraction of WM voxels where the likelihood of ZepZep is higher than that of ZepStick is reported, specifying in how many of those voxels the likelihood ratio test provides a p‐value smaller than 0.05. The histogram shows the overall distribution of p‐values provided by the likelihood ratio test in those voxels where the likelihood of ZepZep is higher than that of ZepStick. The 9 frames in this figure represent subjects (subject 1 in column 1; subject 2 in column 2; subject 3 in column 3) and results from different values of Δ (Δ = 29 ms in row 1; Δ = 52 ms in row 2; Δ = 76 ms in row 3). The figure shows that our data provide limited evidence that accounting for finite intra‐axonal perpendicular improves the quality of fit at the low SNR levels of spinal cord MRI

Figure 7 shows the results from the comparison of the quality of fit of models ZepStick and ZepZep. Model ZepZep provides higher values of likelihood (i.e. better quality of fit) in about three quarters of WM voxels, although in only 1 to 5 percent of those voxels the increased quality of fit cannot be simply justified by the increased number of model parameters (*p* < 0.05 for the likelihood ratio test). Surprisingly, in about a quarter of WM voxels, considering a non‐zero perpendicular intra‐axonal diffusivity has deleterious effects on the quality of fit, as the likelihood of ZepStick surpasses that of ZepZep.

## 4 DISCUSSION

### 4.1 Summary and key results

We studied the diffusion time‐dependence of intra‐/extra‐axonal perpendicular diffusivities (*D*_in, ⊥_, *D*_ex, ⊥_) kurtosis excess (*K*_in, ⊥_, *K*_ex, ⊥_) and of their corresponding DW signals (*S*_in, ⊥_, *S*_ex, ⊥_) with MC simulations, considering geometries representative of spinal cord microstructure (i.e. large axons). Also, we investigated whether time‐dependence can be observed in clinically feasible DW imaging of the spinal cord in vivo, considering well‐established macroscopic DTI and DKI parameters, as well as those from microscopic diffusion anisotropy imaging (microscopic DTI based on SMT), NODDI and two‐compartment SMT.

The key findings from simulation are that in presence of large axons as in the spinal cord, the time‐dependence of the DW signal for clinically feasible acquisitions is dominated by intra‐axonal components. Also, intra‐axonal perpendicular DW signals show considerable attenuation due to the finite axon diameter, which is not modelled in the popular “stick” model for axons. Compartment‐specific kurtosis excess also show time‐dependent patterns, with *K*_in, ⊥_ increasing and *K*_ex, ⊥_ decreasing for clinically achievable diffusion times. Such changes are reflected by the DW signals, whose dependence on diffusion time appears capable of influencing model fitting even in the presence of high noise levels.

In vivo findings highlight diffusion time‐dependence of all metrics considered in this study. Specifically, time‐dependence is a key feature of the DW signal both orthogonally and longitudinally to axons: the often overlooked longitudinal time‐dependence of diffusivity and kurtosis may provide new exciting opportunities for biophysical modelling^19^, ^22^. Lastly, accounting for non‐zero intra‐axonal perpendicular diffusivity improves the quality of fit of biophysically‐inspired models, although only to a minor extent.

### 4.2 In silico study

Simulations highlight time‐dependence of both intra‐axonal and extra‐axonal perpendicular diffusivities and kurtosis excesses in our synthetic model of spinal cord WM, for clinically achievable diffusion times (bigger than roughly 25 ms). Such a time‐dependence is also reflected by the respective intra‐axonal (*S*_in, ⊥_) and extra‐axonal (*S*_ex, ⊥_) perpendicular signals, but to different extents. In particular, *S*_in, ⊥_ varies much more steeply as a function of the diffusion time than *S*_ex, ⊥_, and the latter shows stronger attenuation than the former with the respect to the baseline at *b*=0, in line with previous findings.^21^

The attenuation of the intra‐axonal signal *S*_in, ⊥_ for large axons can be considerable, and up to 40% of the baseline level at *b* = 0. This implies that a popular geometric model for the intra‐axonal compartment, the zero‐radius cylinder (or “stick”),^4^, ^6^, ^36^ may not be the most appropriate for the largest spinal axons, which even if low in number can contribute substantially to the measured signal given their large volume. However, it is likely that such large axons constitute only a fractions of all spinal axons. Therefore, portions of spinal cord WM are likely to behave in a manner that is more in line with our synthetic small axons, as discussed below.

For our synthetic small axons, the “stick” model appears a valid and useful approximation, in line with recent findings.^64^ Time‐dependence for clinically achievable diffusion times originates mainly in the extra‐axonal space, although the simulations also show that the time‐dependent variation of the extra‐axonal signal *S*_ex, ⊥_ is by all means very small. In general, it should be noted that our model of small axons is inspired by callosal WM, and may not be representative of the entire brain. However, while larger axon diameters can indeed be observed in the brain (e.g. higher diameters in the corticospinal tract), they may still be insufficient to cause strong diffusion time‐dependence with clinical hardware.^37^, ^65^ In presence of pathology additional considerations would be needed, as for instance certain diseases such as multiple sclerosis have been reported to induce changes in axon diameters.^66^

Another important observation is related to the tortuosity limit for the extra‐axonal perpendicular diffusivity. The asymptotic value of *D*_ex, ⊥_ provided by simulations is slightly higher than the tortuosity limit that has been adopted in some cases for multi‐compartmental modelling in vivo.^6^, ^11^, ^36^ In vivo, such discrepancies can potentially lead to large fitting errors,^21^ especially given that the elements required to calculate the limit are difficult to estimate. The tortuosity limit is theoretically dependent on the fibre volume fraction FVF and on the intrinsic extra‐axonal diffusivity *D*_ex, 0_ (Equation (4)). However, the estimation of FVF requires orthogonal information on the myelination status of axons,^44^, ^45^, ^67^ since the restricted diffusion signal fraction is essentially equal to AWF, due to the long echo times used in DW MRI. Also, reliable estimation of the intrinsic coefficient *D*_ex, 0_ is known to be extremely error‐prone.^68^ All in all, these considerations suggest that while the tortuosity limit is a first order approximation that can be useful to cope with noisy and scarce data sets, results from analyses that rely on this approximation should be interpreted with extra care.^69^

Our simulation highlight time‐dependence of both intra‐/extra‐axonal perpendicular diffusivities as well as kurtosis excess. Interestingly, the long‐time limit of the intra‐axonal kurtosis excess *K*_in, ⊥_ for both small and large axons are in the leptokurtic regime (well above 0), and thus differ from the platykurtic limit $K_{\text{in},\;⊥}\left(t\to \infty \right)\;=\;-0.5$ for spin displacements within an individual cylinder. This can be explained by the fact that the kurtosis arising from pores (here: cylinders) drawn from a distribution of sizes (here: the gamma distribution of axon radii) can be different from the kurtosis of the individual pore, and carries a signature of the size distribution (here: the long‐time limit of *K*_in, ⊥_ is different for small and large axons).

Importantly, the changes in diffusivity and kurtosis with diffusion time are reflected by changes in the total DW signal for the substrates with large axons. The time‐dependence of the DW signal appears capable of influencing model fitting, since increases in total signal with increasing diffusion time can be detected on average even in presence of high levels of noise, as those typical of spinal cord imaging. For the substrates with small axons the time‐dependence of the total DW signal appears indistinguishable form noise, in line with recent studies that have investigated the sensitivity of DW MRI to axon diameters in the brain.^37^

Finally, the findings discussed above hold in both cases when intra‐axonal water is either faster or slower than extra‐axonal water. This is another key result, since this is still a matter of debate.^14^, ^48^, ^49^, ^63^

### 4.3 In vivo study

We performed clinically feasible DW acquisitions on three subjects using a range of diffusion times (Δ of 29, 52 and 76 ms) and analysed the data to obtain well established macroscopic DTI and DKI metrics, as well as microscopic DTI indices from single‐compartment SMT, NODDI and two‐compartment SMT.

Our results show that it is possible to measure patterns of diffusion time‐dependence of the parameters of clinically viable MR techniques, obtained using a clinical system in the spinal cord. We detect changes that are plausible with current knowledge, i.e. decrease of diffusivity for increasing diffusion time and increases in kurtosis.^20^, ^22^, ^48^ Moreover, we also detect time‐dependence in metrics from popular techniques such as NODDI and two‐compartment SMT. On the one hand, these unexplored patterns of time‐dependence may provide new opportunities for non‐invasive spinal cord microstructural imaging. However, these patterns also denote that at the shortest diffusion times used here, diffusion characteristics do not reach their long‐time limits. This important fact should be considered when adopting analytical constraints based on long‐time approximations of the DW signal, since short diffusion times would be normally adopted to minimise echo time/maximise image quality.

Interestingly, we detect changes in the diffusion charactersitics that are not limited to perpendicular diffusivities, i.e. of axial diffusivity and kurtosis. The change of macroscopic AD_DTI_, AD_DKI_ and AK_DKI_ may be in part due to perpendicular restriction, due to intra‐voxel fibre orientation dispersion.^42^ However, the fact that changes in microscopic DT *μ*AD are also observed, suggests that diffusion restriction can occur longitudinally to axons. While at present the origin of such parallel restrictions is not fully understood, recent research has suggested that they may originate in the intra‐axonal space, from varicosities rich in mitochondria.^22^ Future investigation is needed to confirm and validate similar hypotheses.

In this study we focus on spinal cord WM, considering motor and sensory areas separately. Specifically, we detect time‐dependence of diffusion metrics in both portion of tissues. Interestingly, metrics from NODDI show the same behaviour in the two areas, while two‐compartment SMT metrics show different time‐dependent behaviour in motor compared to sensory WM. This difference between two‐compartment SMT and NODDI, as well as global differences between values of intra‐neurite volume fractions from the two methods (V_NODDI_ and V_SMT_) may be a result of different sensitivities to microstructural heterogeneity, a known feature of spinal cord WM.^70^ Nonetheless, it is also possible that the two techniques have different sensitivity to noise, given their different number and type of model constraints.^6^, ^36^ Future studies in larger cohorts will enable a more accurate comparison of the two methods for spinal cord applications.

Of note, it is known that grey matter (GM) also contains myelinated and unmyelinated axons, for instance from GM interneurons. Therefore, axonal time‐dependence may be a relevant feature also for GM signals. In future, we plan to study in more detail the characteristics of DW signals in spinal cord GM, exploiting improvements in acquisition and hardware that will support a much higher in‐plane and through‐plane resolution.

We have also tested whether accounting for the caliber of axons can prove beneficial for model fitting. Our results show that accounting for departures from the “stick” hypothesis for the intra‐axonal signal provides better quality of fit (i.e. model ZepZep as compared to ZepStick). However, the improvement in goodness of fit is limited and would reach statistical significance only in a small fraction of voxels. The moderate improvement may be a partly due to the intrinsically low quality of our data, lower compared to other studies^53^, ^71^ due to the longer echo time necessary to probe long diffusion times. In any case, we take the opportunity to remark that improved quality of fit is only a necessary but not sufficient condition for better modelling, and therefore model comparisons based on goodness‐of‐fit should alway be taken with care.^72^

Finally, here we observe high value of all axial diffusivities, which exceed values reported in the brain.^73^ This result may be partly due to residual physiological noise^74^ related to cord pulsation^75^ and blood microcirculation,^76^ well known phenomena that make spinal cord DW MRI a challenging task.^31^, ^77^

### 4.4 Limitations

We acknowledge a number of limitations of our approach.

Firstly, our simulations do not model restrictions parallel to the longitudinal axis of axonal segments, and rather focus only on perpendicular restrictions due to axonal membranes. Diffusion restriction parallel to axons appear an important signature of WM DW signals, as shown by our in vivo data and by recent studies in the brain.^22^ However, no well‐established biophysical models that explain this phenomenon exist at present, as recent hypotheses that see its origin in mitochondria need further validation. In future, we will investigate parallel diffusion restriction as well as more realistic axonal shapes and potential transcytolemmal water exchange, in order to further improve the fidelity of our synthetic models of WM.

Secondly, in our simulations we use distributions of axons radii that are inspired by histology, but certainly do not represent the variety of microstructural complexity that characterises spinal cord WM in its entirety. We have studied the effect of deviations from the values assumed here for the parameters of the axon radius distributions, as reported in Supporting Information Figures S3, S4 and S5. The study demonstrates that the results reported in this paper would hold even for considerable variations of the parameters *a* and *b*, and for both cases when intra‐axonal water is either faster or slower than extra‐axonal water. Also, here we used arbitrary values for the intrisic diffusivities of the intra‐/extra‐axonal compartments. We remark that measuring reliably such quantities in vivo is very challenging, and that the exact value of such diffusivities strongly influences quantitative values of model‐based diffusion metrics.

Thirdly, we acknowledge that care is needed when interpreting microscopic DTI metrics from single diffusion encoding, as in this study. Such an acquisition protocol cannot distinguish between distributions of isotropic diffusivities and a spatial distribution of anisotropic diffusivities.^15^, ^63^ However, it should also be noted that macroscopic anisotropy (i.e. differences between AD_DTI_ and RD_DTI_, as detected here) can be observed only in presence of microscopic anisotropy, suggesting that the model used here for the microscopic DT signal is a reasonable choice, at least for healthy WM. Nonetheless, more advanced approaches^15^, ^78^ may enable a better characterisation of the microscopic diffusion signal. Here, we focus on currently clinically viable techniques, and leave such more advanced approaches to future work.

Furthermore, we point out that the multi‐compartment models fitted in vivo are relatively simplistic and do not account for intra‐compartment kurtosis, and thus only allow us to investigate main, first‐order effects. However, at present mapping intra‐compartment kurtosis in vivo is extremely challenging, and the general lower quality of spinal cord DW data as compared to the brain makes this impractical in the clinical scenarios considered here. Here we aim to perform an initial exploratory analysis, and reserve modelling of compartment‐specific higher order cumulants to future work.

Importantly, it should be noted that our in vivo scans are noisier than those that one would perform in a more clinical implementation. Here we investigated specifically the impact of changing diffusion times on popular diffusion metrics from multi‐shell scans, and necessarily needed to adopt a long echo time to achieve 2855 s mm^−2^ with diffusion times of the order of 70 ms, resulting in poorer image quality. It is important to remember that in a real clinical implementation, the same *b*‐values could be probed with much shorter echo times, i.e. obtaining data with higher SNR.

Finally, we point out that the number of subjects of our study is small. Our paper is a first exploratory investigation on the impact of the choice of diffusion time on clinically viable diffusion analyses in the spinal cord. We aim to capture salient effects, and for this purpose we have adopted a robust mixed effects statistical method that allow us to characterise trends with good confidence, by exploiting information from multiple voxels. In future, we aim to study diffusion time‐dependence in a larger cohort of subjects, expanding the preliminary findings reported in this paper.

## 5 CONCLUSIONS

The DW MRI signal of spinal cord WM measured with clinically viable approaches exhibits patterns of diffusion time‐dependence that can be detected with good confidence in vivo. These patterns are reflected by scalar metrics of popular models, and provide new opportunities for non‐invasive microstructure characterisation. Specifically, the time‐dependence of the perpendicular DW signal most likely features substantial intra‐axonal contributions due to large caliber of spinal axons. Therefore, a popular model known as “stick” (zero‐radius cylinder) may be insufficient to describe signals from the largest spinal axons, although model selection tests support this statement only to a limited extent on our noisy in vivo spinal cord data.

## FUNDING

F.G. was funded by the UCL Grand Challenges scheme and is now supported by the Horizon 2020 (H2020) Framework Programme CDS‐QuaMRI grant (634541) and by the Engineering and Physical Sciences Research Council (EPSRC) Platform Grant for medical image computing for next‐generation healthcare technology (EP/M020533/1), through the CMIC Pump‐priming Award. A.I. is funded by the EPSRC grant M507970. C.T. was funded by a 2015 European Committee for Research and Treatment in Multiple Sclerosis (ECTRIMS) post‐doctoral research fellowship. F.P. is supported by a Guarantors of Brain post‐doctoral non‐clinical fellowship. E.K. is funded by the H2020 CDS‐QuaMRI grant (634541). T.S. is an employee of Philips UK. D.C.A. receives research funding from the EPSRC (G007748, I027084, M020533, N018702) and H2020‐EU.3.1 (634541, 666992‐2). C.G.W.K. receives funding from EPSRC (EP/I027084/1), International Spinal Research Trust (UK), Wings for Life (Austria), Craig H. Neilsen Foundation (USA) for the INSPIRED study and H2020‐EU.3.1 (634541). The Queen Square MS Centre is supported by the UK Multiple Sclerosis Society (892/08) and by the Department of Health's National Institute for Health Research Biomedical Research Centres (BRC R&D 03/10/RAG0449).

## Supporting information

Supporting Information fo Figures
