# Supplementary material for: Relevance of time‐dependence for clinically viable diffusion imaging of the spinal cord
Source: Magn Reson Med. 2018 Sep 5;81(2):1247–64. doi: 10.1002/mrm.27463 (PMC6586052; doi:10.1002/mrm.27463)
Supplement: Supplementary file 1 — Supporting Information fo Figures [file MRM-81-1247-s001.pdf]

Supporting Information Figure S1: simulations for

$$D_{\mathrm{in},0} = 1\ \mu\mathrm{m}^2\,\mathrm{ms}^{-1}$$
$$D_{\mathrm{ex},0} = 2\ \mu\mathrm{m}^2\,\mathrm{ms}^{-1}$$

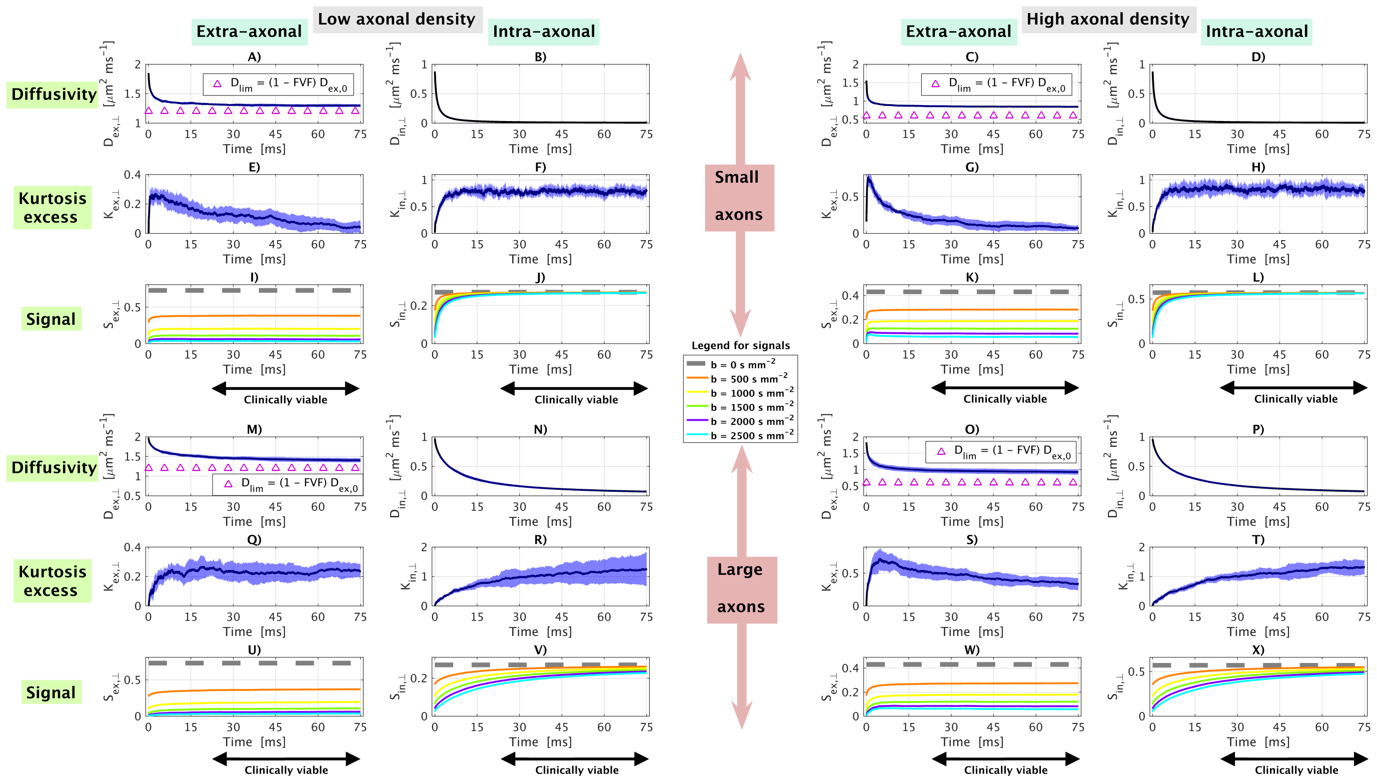

Supporting Information Figure S1: time-dependent perpendicular diffusivities, kurtosis excess and DW signals characterising the intra-axonal and extra-axonal compartments when $D_{\mathrm{ex},0} = 2\,\mu\mathrm{m}^2\,\mathrm{ms}^{-1}$ and $D_{\mathrm{in},0} = 1\,\mu\mathrm{m}^2, \mathrm{ms}^{-1}$ (i.e. extra-axonal water is faster than intra-axonal water). The first two columns from the left refer to substrates with low axonal densities (extra-axonal properties in panels A, E, I, M, Q, U; intra-axonal properties in panels B, F, J, N, R, V), while the last two columns from the left refer to substrates with high axonal density (extra-axonal properties in panels C, G, K, O, S, W; intra-axonal properties in panels D, H, L, P, T, X). The first three rows from top show information for substrates with small axons (A to L), while the last three rows from top show information for substrates with large axons (M to X). The first and fourth rows from top show time-dependent diffusivities (A to D and M to P), with the tortuosity limit $D_{\mathrm{lim}} = (1 - \mathrm{FVF})\, D_{\mathrm{ex},0}$ reported explicitly for extra-axonal diffusivities (magenta triangles in A, C, M, O); the second and fifth rows from top show time-dependent kurtosis excess (E to H and Q to T); the third and sixth rows provide DW signals (I to L and U to X). The plots of diffusivities and kurtosis excess report mean and standard deviation over the 11 random seeds respectively in black and light blue shade. The plots of the DW signals report signals obtained at different b-values, obtained using the average diffusivity and kurtosis excess over the random seeds. Black arrows indicate diffusion times that can be probed with clinically viable acquisitions.

Supporting Information Figure S2: simulations for

$$D_{\mathrm{in},0} = 1\ \mu\mathrm{m}^2\,\mathrm{ms}^{-1}$$
$$D_{\mathrm{ex},0} = 2\ \mu\mathrm{m}^2\,\mathrm{ms}^{-1}$$

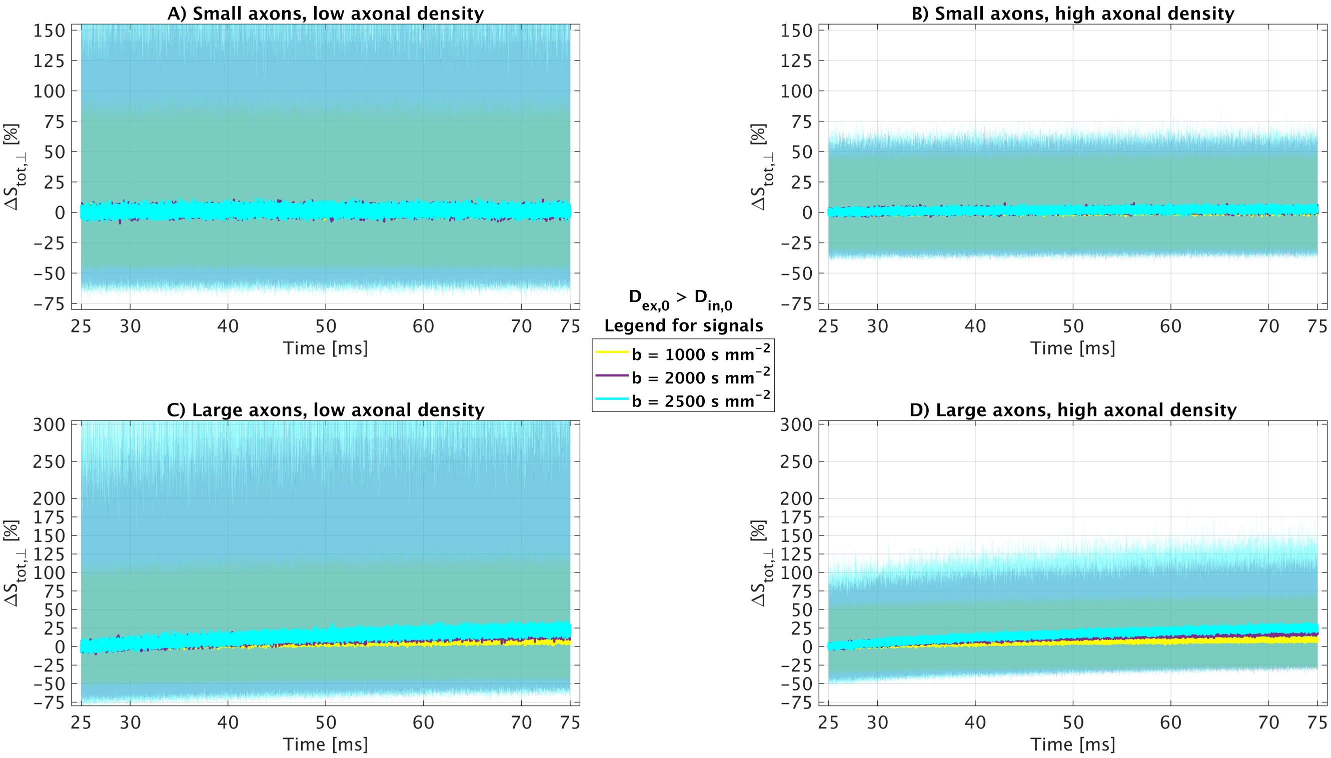

Supporting Information Figure S2: time-dependent patterns of $\Delta S_{\mathrm{tot},\perp}(t\,,\,b)$ [%], defined as the percentage relative differences between the total DW signal at a diffusion time t with respect to the total signal at a reference diffusion time $t = t_{\mathrm{ref}} = 25\,\mathrm{ms}$. The figure reports in yellow, violet and cyan values of $\Delta S_{\mathrm{tot},\perp}(t\,,\,b)$ [%] obtained respectively at $b = \{1000,\,2000,\,2500\}\ \mathrm{s\,mm}^{-2}$, for the four synthetic substrates (small/large axons; low/high axonal density) when $D_{\mathrm{ex},0} = 2\,\mu\mathrm{m}^2\,\mathrm{ms}^{-1}$ and $D_{\mathrm{in},0} = 1\,\mu\mathrm{m}^2, \mathrm{ms}^{-1}$ (i.e. extra-axonal water is faster than intra-axonal water). Solid lines report median values over 500 independent noise realisations, with 95% of the distributions over the realisations reported as a transparent background on beneath.

Supporting Information Figure S3: sensitivity of simulations to the choice of the axon radius distribution

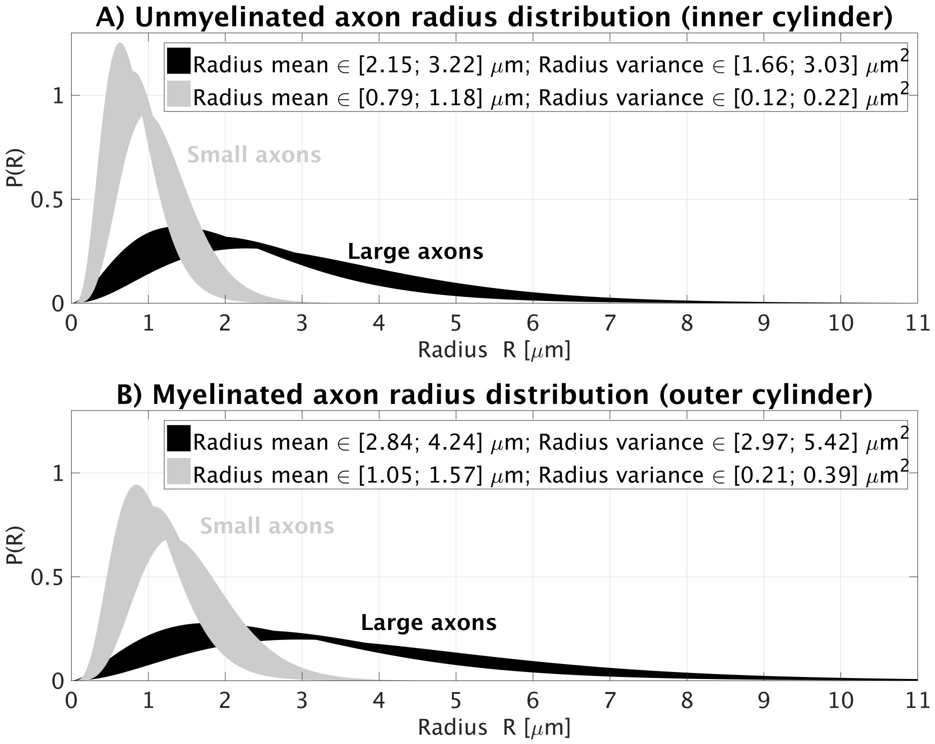

Supporting Information Figure S3: axon radius distributions probed by the sensitivity analysis, where the scale and shape parameters (a and b) of the gamma distributions describing axon radii have been perturbed of $\pm$ 10 % from their nominal values reported in the main article. The figure shows distributions for the unmyelinated axons (i.e. inner cylinders; panel A, on top) as well as for the for the myelinated axons (i.e. outer cylinders; panel B, to the bottom). Black curves report information for the large axons, while grey curves for the small axons. The legends report the corresponding ranges for radius mean value ($E[R] = a\,b$) and variance ($Var[R] = a\,b^2$).

Supporting Information Figure S4: sensitivity of simulations to the choice of the axon radius distribution

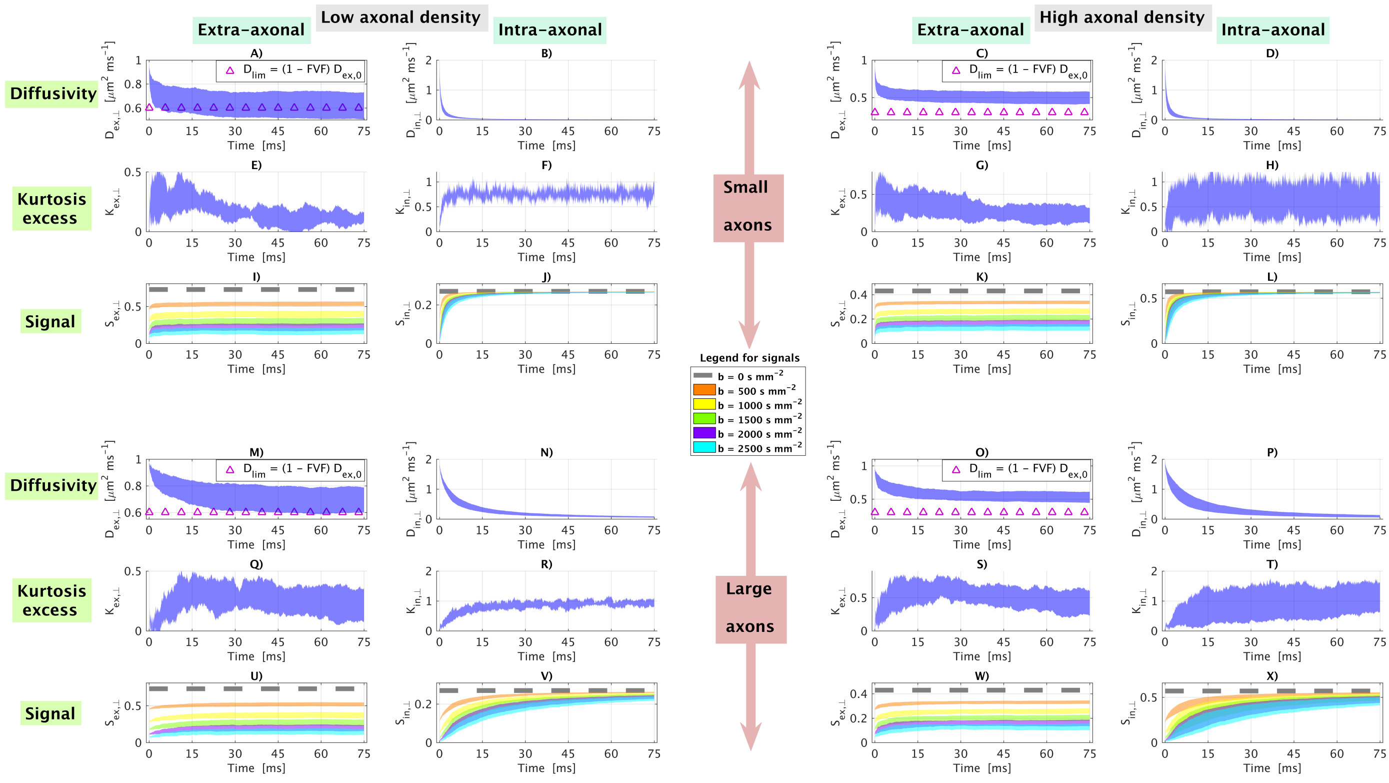

Supporting Information Figure S4: time-dependent perpendicular diffusivities, kurtosis excess and DW signals characterising the intra-axonal and extra-axonal compartments when $D_{\text{ex},0} = 1\,\mu\text{m}^2\,\text{ms}^{-1}$ and $D_{\text{in},0} = 2\,\mu\text{m}^2\,\text{ms}^{-1}$ (i.e. intra-axonal water is faster than extra-axonal water) for perturbations of the axon radius distributions. The first two columns from the left refer to substrates with low axonal densities (extra-axonal properties in panels A, E, I, M, Q, U; intra-axonal properties in panels B, F, J, N, R, V), while the last two columns from the left refer to substrates with high axonal density (extra-axonal properties in panels C, G, K, O, S, W; intra-axonal properties in panels D, H, L, P, T, X). The first three rows from top show information for substrates with small axons (A to L), while the last three rows from top show information for substrates with large axons (M to X). The first and fourth rows from top show time-dependent diffusivities (A to D and M to P), with the tortuosity limit $D_{\text{lim}} = \left(1 - \text{FVF}\right) D_{\text{ex},0}$ reported explicitly for extra-axonal diffusivities (magenta triangles in A, C, M, O); the second and fifth rows from top show time-dependent kurtosis excess (E to H and Q to T); the tird and sixth rows provide DW signals (I to L and U to X). The plots of diffusivities, kurtosis excess and signals report the range obtained over the perturbations. The perturbations were obtained using the same simulation settings described in the main manuscript for one fixed random seed, but perturbing the values of a and b (parameters of the gamma distributions) of $\pm$ 10 %. The reference values of (a,b) that were perturbed were: $a = 3.01$, $b = 1.63\,\mu$m for the outer cylinder and $a = 3.11$, $b = 0.86\,\mu$m for the inner cylinder (substrate with large axons); $a = 5.73$ and $b = 0.23\,\mu$m for the outer cylinder and $a = 5.69$ and $b = 0.17\,\mu$m for the inner cylinder (substrate with small axons).

Supporting Information Figure S5: sensitivity of simulations to the choice of the axon radius distribution

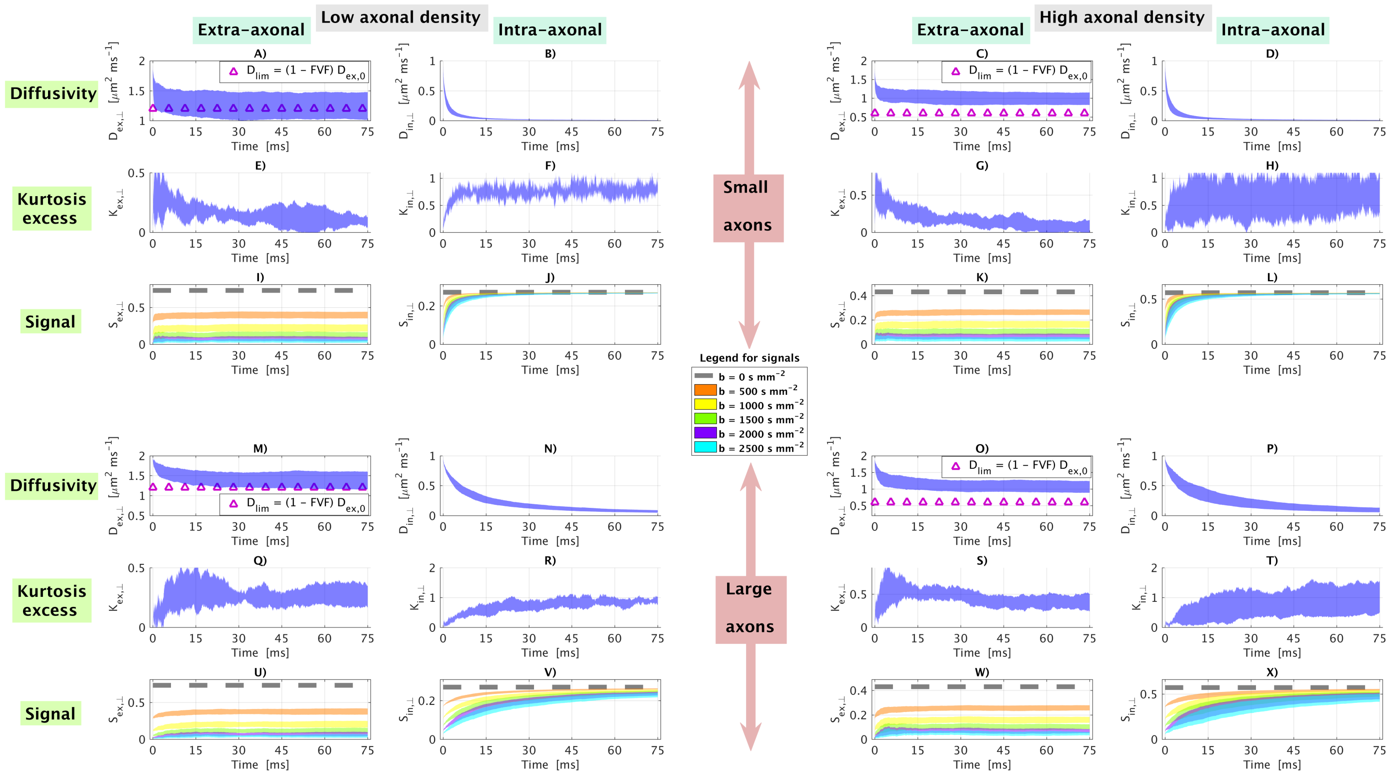

Supporting Information Figure S5: time-dependent perpendicular diffusivities, kurtosis excess and DW signals characterising the intra-axonal and extra-axonal compartments when $D_{\mathrm{ex},0} = 2\,\mu\mathrm{m}^2\,\mathrm{ms}^{-1}$ and $D_{\mathrm{in},0} = 1\,\mu\mathrm{m}^2\,\mathrm{ms}^{-1}$ (i.e. intra-axonal water is slower than extra-axonal water) for perturbations of the axon radius distributions. The first two columns from the left refer to substrates with low axonal densities (extra-axonal properties in panels A, E, I, M, Q, U; intra-axonal properties in panels B, F, J, N, R, V), while the last two columns from the left refer to substrates with high axonal density (extra-axonal properties in panels C, G, K, O, S, W; intra-axonal properties in panels D, H, L, P, T, X). The first three rows from top show information for substrates with small axons (A to L), while the last three rows from top show information for substrates with large axons (M to X). The first and fourth rows from top show time-dependent diffusivities (A to D and M to P), with the tortuosity limit $D_{\mathrm{lim}} = \left(1 - \mathrm{FVF}\right) D_{\mathrm{ex},0}$ reported explicitly for extra-axonal diffusivities (magenta triangles in A, C, M, O); the second and fifth rows from top show time-dependent kurtosis excess (E to H and Q to T); the tird and sixth rows provide DW signals (I to L and U to X). The plots of diffusivities, kurtosis excess and signals report the range obtained over the perturbations. The perturbations were obtained using the same simulation settings described in the main manuscript for one fixed random seed, but perturbing the values of a and b (parameters of the gamma distributions) of $\pm$ 10 %. The reference values of (a,b) that were perturbed were: $a = 3.01$, $b = 1.63\,\mu$m for the outer cylinder and $a = 3.11$, $b = 0.86\,\mu$m for the inner cylinder (substrate with large axons); $a = 5.73$ and $b = 0.23\,\mu$m for the outer cylinder and $a = 5.69$ and $b = 0.17\,\mu$m for the inner cylinder (substrate with small axons).
